# Exploring the diversity of *Diplostomum* (Digenea: Diplostomidae) in fishes from the River Danube using mitochondrial DNA barcodes

**DOI:** 10.1186/s13071-017-2518-5

**Published:** 2017-12-02

**Authors:** Olena Kudlai, Mikuláš Oros, Aneta Kostadinova, Simona Georgieva

**Affiliations:** 1 Water Research Group, Unit for Environmental Sciences and Management, Potchefstroom Campus, North-West University, Potchefstroom, 2520 South Africa; 2 Institute of Parasitology, Biology Centre of the Czech Academy of Sciences, Branišovská 31, 370 05 České Budějovice, Czech Republic; 3 Institute of Ecology, Nature Research Centre, Akademijos 2, 08412 Vilnius, Lithuania; 4 Institute of Parasitology, Slovak Academy of Sciences, Hlinkova 3, 040 01 Košice, Slovak Republic

**Keywords:** *Diplostomum*, Diplostomidae, Metacercariae, Freshwater fishes, Barcodes, *cox*1, *nad*3, River Danube, Europe

## Abstract

**Background:**

Metacercariae of *Diplostomum* are important fish pathogens, but reliable data on their diversity in natural fish populations are virtually lacking. This study was conducted to explore the species diversity and host-parasite association patterns of *Diplostomum* spp. in a large riverine system in Europe, using molecular and morphological data.

**Methods:**

Twenty-eight species of fish of nine families were sampled in the River Danube at Nyergesújfalu in Hungary in 2012 and Štúrovo in Slovakia in 2015. Isolates of *Diplostomum* spp. were characterised morphologically and molecularly. Partial sequences of the ‘barcode’ region of the cytochrome *c* oxidase subunit 1 (*cox*1) and complete sequences of the nicotinamide adenine dinucleotide dehydrogenase subunit 3 (*nad*3) mitochondrial genes were amplified for 76 and 30 isolates, respectively. The partial *cox*1 sequences were used for molecular identification of the isolates and an assessment of haplotype diversity and possible host-associated structuring of the most prevalent parasite species. New primers were designed for amplification of the mitochondrial *nad*3 gene.

**Results:**

Only lens-infecting *Diplostomum* spp. were recovered in 16 fish species of five families. Barcoding of representative isolates provided molecular identification for three species/species-level genetic lineages, *D*. *spathaceum*, *D*. *pseudospathaceum* and ‘*D*. *mergi* Lineage 2’, and three single isolates potentially representing distinct species. Molecular data helped to elucidate partially the life-cycle of ‘*D*. *mergi* Lineage 2’. Many of the haplotypes of *D*. *spathaceum* (16 in total), *D*. *pseudospathaceum* (15 in total) and ‘*D*. *mergi* Lineage 2’ (7 in total) were shared by a number of fish hosts and there was no indication of genetic structuring associated with the second intermediate host. The most frequent *Diplostomum* spp. exhibited a low host-specificity, predominantly infecting a wide range of cyprinid fishes, but also species of distant fish families such as the Acipenseridae, Lotidae, Percidae and Siluridae. The *nad*3 gene exhibited distinctly higher levels of interspecific divergence in comparison with the *cox*1 gene.

**Conclusions:**

This first exploration of the species diversity and host ranges of *Diplostomum* spp., in natural fish populations in the River Danube, provided novel molecular, morphological and host-use data which will advance further ecological studies on the distribution and host ranges of these important fish parasites in Europe. Our results also indicate that the *nad*3 gene is a good candidate marker for multi-gene approaches to systematic estimates within the genus.

**Electronic supplementary material:**

The online version of this article (10.1186/s13071-017-2518-5) contains supplementary material, which is available to authorized users.

## Background

Metacercariae of the genus *Diplostomum* von Nordmann, 1832 (Digenea: Diplostomidae) are important fish pathogens [1–3] and represent a case study illustrating the difficulties of species identification based solely on morphological data. The recent use of molecular markers proved to be a valuable and efficient approach to species delimitation and identification, especially for the larval stages of *Diplostomum* spp. which lack reliable distinguishing morphological characters. Recent intensive molecular studies, following the publication of the genus-specific primers for the ‘barcode’ region of the cytochrome *c* oxidase subunit 1 (*cox*1) gene [4], resulted in the generation of sequence libraries for the North American [5, 6] and European species [3, 7–12] of the genus. Thus providing a sound basis for molecular identification and provisional species delineation. These libraries provide a foundation that will allow identification of life-cycle stages and ensure an increased taxonomic resolution in epidemiological and ecological studies of these important fish parasites (e.g. Locke et al. [13]; Désilets et al. [14]; Pérez-del-Olmo et al. [3]) as well as for further exploration of species host and geographical ranges [6].

To date, molecular data for a total of 19 species/species-level genetic lineages of *Diplostomum* exist from North America including three named species, i.e. *Diplostomum baeri* Dubois, 1937, *Diplostomum huronense* (La Rue, 1927) and *Diplostomum indistinctum* (Guberlet, 1923), and 16 otherwise unidentified species or species-level lineages [4–6, 15]. Extensive studies carried out in Europe recently revealed a total of 12 species/species-level genetic lineages including two species complexes: *D*. *spathaceum* (Rudolphi, 1819); *D*. *pseudospathaceum* Niewiadomska, 1984; *D*. *parviventosum* Dubois, 1932; three species-level lineages within the “*D*. *baeri*” species complex (*Diplostomum* sp. ‘Lineages 3–5’ *sensu* Blasco-Costa et al., 2014 [9]); three species-level lineages within the “*D*. *mergi*” species complex (*Diplostomum* sp. ‘Lineages 2–4’ *sensu* Georgieva et al., 2013 [7] and Selbach et al., 2015 [10]); *Diplostomum* sp. ‘Clade Q’ *sensu* Georgieva et al., 2013 [7]; and *Diplostomum* sp. ‘Lineages 2 and 6’ *sensu* Blasco-Costa et al., 2014 [9] (see [3, 7, 9, 10, 12, 16]).

However, although molecular data for metacercariae of *Diplostomum* spp. in fishes from European freshwater ecosystems have accumulated recently, most of the sequences originate from fish populations sampled in ponds and lakes in central and northern Europe (Germany, Iceland, Norway), and also predominantly from salmonid fishes. A single study provided molecular and morphological data for metacercariae of three species of *Diplostomum* spp. in endemic and invasive fish host species in Spain, at the southern distributional range of *Diplostomum* spp. in Europe [3]. However, no molecular data exist on species diversity and host ranges of these fish pathogens in large river systems in Europe.

Our study is the first to explore species diversity and host-parasite association patterns of *Diplostomum* spp. in a large riverine system in Europe. Here we extend the *cox*1 ‘barcode’ reference library for *Diplostomum* spp. based on an extensive sampling of metacercariae from a broad range of fish hosts collected at two localities in the middle section of the River Danube. We provide molecular identification based on the *cox*1 gene in association with a thorough morphological characterisation of the metacercariae. Further, we provide primers and the first assessment of the usefulness of the mitochondrial nicotinamide adenine dinucleotide dehydrogenase subunit 3 (*nad*3) gene for species delineation within *Diplostomum* spp.

## Methods

### Sample collection and processing

A total of 174 fish belonging to 28 species of 9 families were sampled in the River Danube near Nyergesújfalu (47.7658N, 18.5417E) in Hungary in 2012 and at Štúrovo (47.8197N, 18.7286E) in Slovakia in 2015. As a part of a complete helminthological examination, fish eyes and brains were isolated and examined for the presence of metacercariae of *Diplostomum* spp. The eyes were dissected and lens, vitreous humour and retina were placed in 0.9% saline solution and examined under a dissecting microscope. All metacercariae were collected and counted. Representative subsamples were selected for DNA isolation and sequencing.

### Morphological examination

The morphology of the metacercariae selected for sequencing was initially studied in live parasites; these were then transferred to molecular grade ethanol and re-examined. A series of photomicrographs was made for each isolate (live and fixed) using a digital camera of an Olympus BX51 microscope (Olympus Corporation, Tokyo, Japan). Measurements for each isolate were taken from the digital images with the aid of Quick Photo Camera 2.3 image analysis software. All measurements in the descriptions and tables are in micrometres and are presented as the range, followed by the mean in parentheses.

Fourteen morphometric variables were measured from the digital images of live and fixed metacercariae and the number of excretory concretions was recorded from live material. The following abbreviations for variables were used: BL, body length; BW, body width; HL, hindbody length; OSL, oral sucker length; OSW, oral sucker width; PSL, pseudosucker length; PSW, pseudosucker width; VSL, ventral sucker length; VSW, ventral sucker width; PHL, pharynx length; PHW, pharynx width; HOL, holdfast organ length; HOW, holdfast organ width; AVS, distance from anterior extremity of body to ventral sucker.

### Sequence generation

Genomic DNA (gDNA) was isolated from single metacercariae using the E.Z.N.A. Tissue DNA Kit (Omega Bio-tek, Norcross, USA) following the manufacturer’s instructions. Amplification of the mitochondrial (mt) *cox*1 gene was performed with the forward primer Plat-diploCOX1F (5′-CGT TTR AAT TAT ACG GAT CC-3′) and the reverse primer Plat-diploCOX1R (5′-AGC ATA GTA ATM GCA GCA GC-3′) [4]. A pair of newly designed primers was used for amplification of the complete *nad*3 mt gene: forward Diplo-nad3F (5′-ATG TGA AAG TGG TGT TTG TT-3′) and reverse Diplo-nad3R (5′-ATG CGC TTA TGA TCT AAC GT-3′). PCR amplifications for both genes were performed in a total volume of 20 μl (8 pmol of each primer) with *c.*50 ng of gDNA and 10 μl of 2× MyFi™ DNA Polymerase mix (Bioline Inc., Taunton, USA). Thermocycling started with an initial DNA denaturation for 2 min at 94 °C followed by 35 cycles with 30 s DNA denaturation at 94 °C, 30 s primer annealing at 50 °C for *cox*1 (57 °C for *nad*3), and 60 s at 72 °C for primer extension, followed by a final extension step of 10 min at 72 °C. PCR amplicons were purified using a QIAquick PCR purification kit (Qiagen Ltd., Hilden, Germany). Cycle sequencing of purified DNA was carried out using ABI Big Dye™ chemistry (ABI Perkin-Elmer, London, UK) on an Applied Biosystems 3730xl DNA Analyser following the manufacturer’s recommendations, using the primers used for PCR amplification. Contiguous sequences were assembled with MEGA v6 [17] and submitted to GenBank under accession numbers KY653961–KY654066.

Unique *cox*1 haplotypes were identified with DnaSP [18] against all published sequences for a given species/lineage. Unrooted statistical parsimony haplotype networks were constructed for *D. spathaceum* and *D. pseudospathaceum* using TCS 1.21 [19] with plausible branch connections between the haplotypes at a connection limit of 95% [20].

### Phylogenetic analyses

Sequences were aligned using MUSCLE implemented in MEGA v6. Two alignments were analysed. The *cox*1 alignment (410 nt) comprised 76 newly generated sequences and 31 sequences for *Diplostomum* spp. retrieved from GenBank; *Tylodelphys clavata* (von Nordmann, 1832) was used as the outgroup. The *nad*3 alignment (357 nt) comprised 30 newly generated sequences and two published sequences, *D*. *pseudospathaceum* and *D*. *spathaceum*. Both alignments included no insertions or deletions and were aligned with reference to the amino acid translation, using the echinoderm and flatworm mitochondrial code [21]. Distance-based neighbour-joining (NJ) and model-based Bayesian inference (BI) algorithms were conducted to identify and explore relationships among the species/isolates. Neighbour-joining analyses of Kimura 2-parameter distances were carried out using MEGA v6; nodal support was estimated using 1000 bootstrap resamplings. Bayesian inference analysis was performed for the *cox*1 dataset using MrBayes version 3.2.3 [22]. Prior to BI analysis, the best-fit nucleotide substitution model was selected in jModelTest 2.1.1 [23] using the Akaike Information Criterion (AIC). This was the general time reversible model, with estimates of invariant sites and gamma distributed among-site rate variation (GTR + I + Г). BI analysis was run with the following nucleotide substitution model settings: lset nst = 6, rates = invgamma, samplefreq = 100, ncat = 4, shape = estimate, inferrates = yes and basefreq = empirical. Markov chain Monte Carlo (MCMC) chains were run for 10,000,000 generations, log-likelihood scores were plotted and only the final 75% of trees were used to produce the consensus trees by setting the ‘burn-in’ parameter at 2500. Results were visualised in Tracer v.1.6 (http://tree.bio.ed.ac.uk/software/tracer/) to assess convergence and proper sampling and to identify the ‘burn-in’ period.

Distance matrices (uncorrected p-distance model) were calculated with MEGA v6. The nomenclature of Georgieva et al. [7] for the lineages of *Diplostomum* spp. was applied for consistency with previous records.

## Results

### General observations

A total of 174 fish individuals belonging to 28 species and 9 families were examined for the presence of metacercariae of *Diplostomum* spp. in the eyes and brain. Only lens-infecting metacercariae were found in 16 fish species of 5 families: 12 cyprinids, one acipenserid, one lotid, one percid and one silurid (Table 1). The overall *Diplostomum* spp. intensity of infection was low (1–15 metacercariae per fish) with two exceptions: *Abramis brama* (25–43, four fishes) and *Blicca bjoerkna* (27, one fish). The overall *Diplostomum* spp. prevalence appeared rather high in five cyprinids (*Leuciscus aspius*: 89%; *Vimba vimba*: 89%; *A. brama*: 83%; *B. bjoerkna*: 77%; and *Alburnus alburnus*: 57%) but reliable estimates for prevalence could be obtained only for the sample of *A. brama*. In this sample, the prevalence of three species/lineages identified in our study (see below) was high: *D. spathaceum*: 75%; ‘*D*. *mergi* Lineage 2’: 58%; *D. pseudospathaceum*: 50%. Twelve species of fish, for which fewer specimens were examined, were not infected.

**Table 1:** Summary data for the fish species examined/infected with *Diplostomum* spp.

Host species	No. examined	No. infected	*Diplostomum* spp.
Acipenseridae			
*Acipenser ruthenus* L.	1	1	*D*. *spathaceum*
Anguillidae			
*Anguilla anguilla* (L.)	1	–	–
Centrarchidae			
*Lepomis gibbosus* (L.)	11	–	–
Cyprinidae			
*Abramis brama* (L.)	41	34	*D. spathaceum*, *D. pseudospathaceum*, ‘*D. mergi* Lineage 2’
*Alburnus alburnus* (L.)	7	4	‘*D. mergi* Lineage 2’
*Ballerus sapa* (Pallas)	9	2	*D. pseudospathaceum*, ‘*D. mergi* Lineage 2’
*Blicca bjoerkna* (L.)	13	10	*D. spathaceum*, *D. pseudospathaceum*, ‘*D. mergi* Lineage 2’, *Diplostomum* sp. A
*Carassius gibelio* (Bloch)	6	1	*Diplostomum* sp. B
*Chondrostoma nasus* (L.)	11	4	*D. spathaceum*, ‘*D. mergi* Lineage 2’
*Cyprinus carpio* L.	3	1	*D. pseudospathaceum*
*Leuciscus aspius* (L.)	9	8	*D. spathaceum*, *D. pseudospathaceum*
*Leuciscus idus* (L.)	4	1	*D. pseudospathaceum*
*Rutilus pigus* (Lacépède)	3	2	*D. spathaceum*
*Rutilus rutilus* (L.)	9	4	*D. spathaceum*, *Diplostomum* sp. C
*Vimba vimba* (L.)	9	8	*D. spathaceum*, *D. pseudospathaceum*, ‘*D. mergi* Lineage 2’
*Barbus barbus* (L.)	2	–	–
*Gobio gobio* (L.)	6	–	–
Esocidae			
*Esox lucius* L.	3	–	–
Gobiidae			
*Neogobius melanostomus* (Pallas)	8	–	–
*Ponticola kessleri* (Günther)	2	–	–
Lotidae			
*Lota lota* (L.)	2	1	*D. pseudospathaceum*
Percidae			
*Gymnocephalus schraetser* (L.)	5	1	*D. pseudospathaceum*
*Perca fluviatilis* L.	3	–	–
*Sander lucioperca* (L.)	1	–	–
*Sander volgensis* (Gmelin)	2	–	–
*Zingel zingel* (L.)	1	–	–
*Zingel streber* (Siebold)	1	–	–
Siluridae			
*Silurus glanis* L.	1	1	*D. spathaceum*

### Molecular identification, haplotype diversity and host-use

**Fig. 1:**
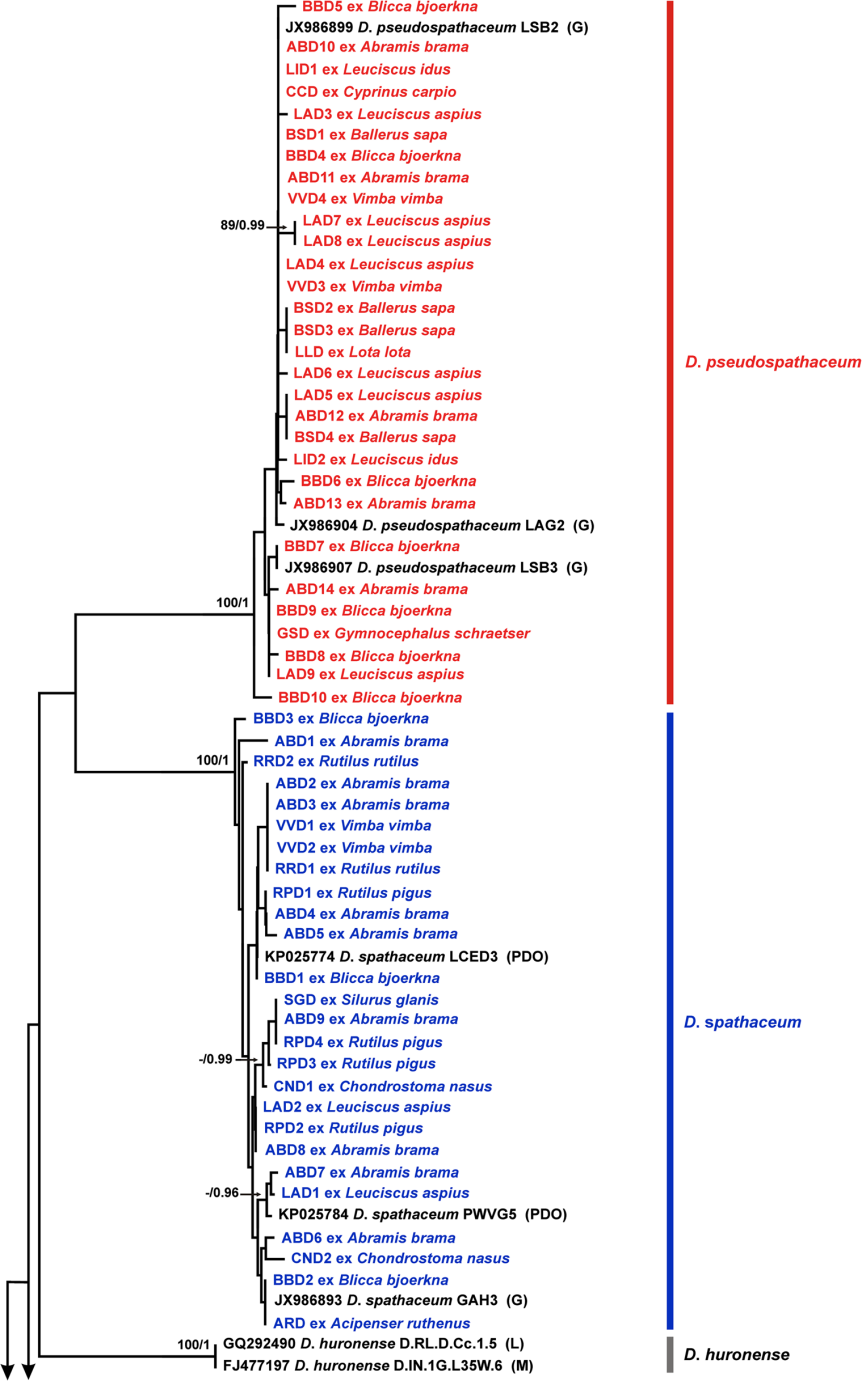
Neighbour-joining (NJ) phylogram for *Diplostomum* spp. reconstructed using 76 newly generated and 31 *cox*1 sequences retrieved from GenBank. Outgroup: *Tylodelphys clavata*. Nodal support from NJ and Bayesian inference (BI) analyses are indicated as NJ/BI; only values > 70% (NJ) and > 0.95 (BI) are shown. The scale-bar indicates the expected number of substitutions per site. Codes for the newly sequenced isolates are provided in Table 2. Sequence identification is as in GenBank, followed by a letter: G, Georgieva et al. [7]; L, Locke et al. [5]; M, Moszczynska et al. [4]; PDO, Pérez-del-Olmo et al. [3]

**Fig. 2:**
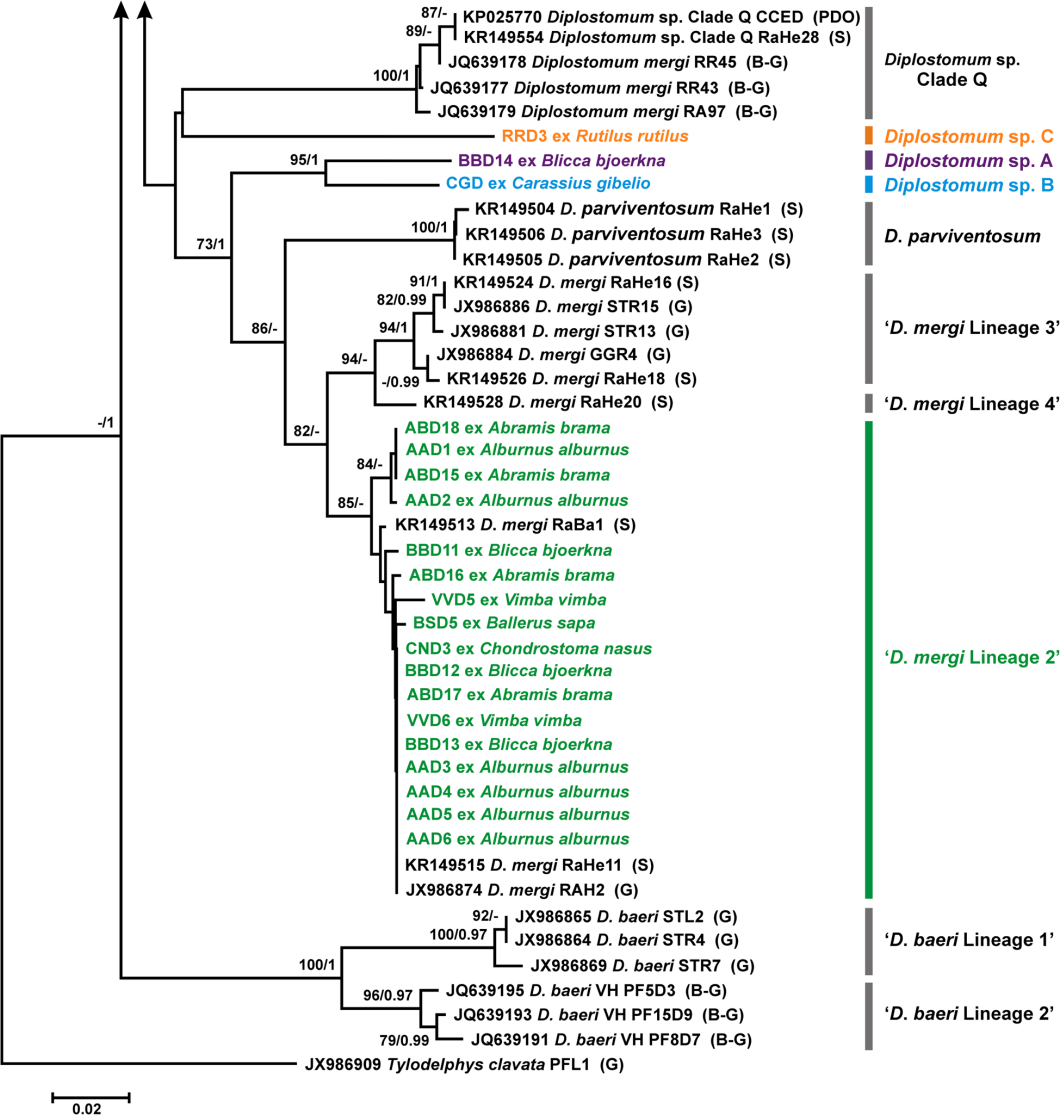
Neighbour-joining (NJ) phylogram for *Diplostomum* spp. reconstructed using 76 newly generated and 31 *cox*1 sequences retrieved from GenBank; continuation of Fig. 1. Nodal support from NJ and Bayesian inference (BI) analyses are indicated as NJ/BI; only values > 70% (NJ) and > 0.95 (BI) are shown. The scale-bar indicates the expected number of substitutions per site. Codes for the newly sequenced isolates are provided in Table 2. Sequence identification is as in GenBank, followed by a letter: B-G, Behrmann-Godel [8]; G, Georgieva et al. [7]; PDO, Pérez-del-Olmo et al. [3]; S, Selbach et al. [10]

We generated partial *cox*1 sequences (410 nt) for 76 isolates of *Diplostomum* spp. recovered from fishes of the River Danube (Table 2). These sequences were analysed together with 31 sequences for 10 *Diplostomum* species/species-level genetic lineages retrieved from the GenBank database (see Additional file 1: Table S1 for details). All lens-infecting species/lineages of *Diplostomum* (7) reported in Europe were included in analyses: *D*. *parviventosum*, *D*. *pseudospathaceum*, *D*. *spathaceum*, ‘*D. mergi* Lineage 2’, ‘*D. mergi* Lineage 3’, ‘*D. mergi* Lineage 4’, ‘*Diplostomum* sp. Clade Q’ *sensu* Georgieva et al., 2013 [7]. We also included sequences for *D*. *huronense* (a species believed to have a Holarctic distribution; see [24]) and two representatives of non-lens infecting species of the “*D. baeri*” complex. The branch topologies of the trees resulting from both, NJ and BI analyses, were in consensus in depicting species/species-level genetic lineages (Figs. 1, 2). The newly generated sequences clustered within three well-supported clades representing *D*. *pseudospathaceum*, *D*. *spathaceum* and ‘*D. mergi* Lineage 2’ except for three singletons which may potentially represent distinct species (labelled as *Diplostomum* sp. A, B and C in Fig. 2). Two of these (*Diplostomum* sp. A and B) were resolved as basal to the clade representing the “*D. mergi*” species complex, whereas *Diplostomum* sp. C appeared associated with ‘Clade Q’; however, these relationships were not supported.

**Table 2:** Summary data for the isolates of *Diplostomum* spp. used for generation of the *cox*1 and *nad*3 sequences

Species	Host	Country	Isolate	Haplotype (*cox*1)	GenBank ID	
					*cox*1	*nad*3
*D. spathaceum*	*Abramis brama*	S	ABD1	H11	KY653961	KY654037
*D. spathaceum*	*Abramis brama*	S	ABD2	H1	KY653962	
*D. spathaceum*	*Abramis brama*	S	ABD3	H1	KY653963	
*D. spathaceum*	*Abramis brama*	S	ABD4	H5	KY653964	
*D. spathaceum*	*Abramis brama*	S	ABD5	H9	KY653965	
*D. spathaceum*	*Abramis brama*	S	ABD6	H12	KY653966	KY654038
*D. spathaceum*	*Abramis brama*	S	ABD7	H10	KY653967	
*D. spathaceum*	*Abramis brama*	S	ABD8	H2	KY653968	
*D. spathaceum*	*Abramis brama*	S	ABD9	H3	KY653969	KY654039
*D. spathaceum*	*Acipenser ruthenus*	S	ARD	H4	KY653970	
*D. spathaceum*	*Blicca bjoerkna*	S	BBD1	H6	KY653971	
*D. spathaceum*	*Blicca bjoerkna*	S	BBD2	H4	KY653972	KY654040
*D. spathaceum*	*Blicca bjoerkna*	H	BBD3	H14	KY653973	
*D. spathaceum*	*Chondrostoma nasus*	S	CND1	H7	KY653974	KY654041
*D. spathaceum*	*Chondrostoma nasus*	H	CND2	H15	KY653975	
*D. spathaceum*	*Leuciscus aspius*	H	LAD1	H13	KY653976	KY654042
*D. spathaceum*	*Leuciscus aspius*	S	LAD2	H2	KY653977	
*D. spathaceum*	*Rutilus pigus*	S	RPD1	H5	KY653978	
*D. spathaceum*	*Rutilus pigus*	S	RPD2	H2	KY653979	KY654043
*D. spathaceum*	*Rutilus pigus*	S	RPD3	H8	KY653980	
*D. spathaceum*	*Rutilus pigus*	S	RPD4	H3	KY653981	KY654044
*D. spathaceum*	*Rutilus rutilus*	S	RRD1	H1	KY653982	KY654045
*D. spathaceum*	*Rutilus rutilus*	H	RRD2	H16	KY653983	
*D. spathaceum*	*Silurus glanis*	S	SGD	H3	KY653984	KY654046
*D. spathaceum*	*Vimba vimba*	S	VVD1	H1	KY653985	
*D. spathaceum*	*Vimba vimba*	S	VVD2	H1	KY653986	
*D. pseudospathaceum*	*Abramis brama*	S	ABD10	H1	KY653987	KY654047
*D. pseudospathaceum*	*Abramis brama*	S	ABD11	H1	KY653988	
*D. pseudospathaceum*	*Abramis brama*	S	ABD12	H2	KY653989	KY654048
*D. pseudospathaceum*	*Abramis brama*	S	ABD13	H14	KY653990	
*D. pseudospathaceum*	*Abramis brama*	S	ABD14	H15	KY653991	
*D. pseudospathaceum*	*Ballerus sapa*	S	BSD1	H1	KY653992	KY654049
*D. pseudospathaceum*	*Ballerus sapa*	S	BSD2	H3	KY653993	KY654050
*D. pseudospathaceum*	*Ballerus sapa*	S	BSD3	H3	KY653994	
*D. pseudospathaceum*	*Ballerus sapa*	S	BSD4	H2	KY653995	
*D. pseudospathaceum*	*Blicca bjoerkna*	H	BBD4	H1	KY653996	
*D. pseudospathaceum*	*Blicca bjoerkna*	S	BBD5	H7	KY653997	KY654051
*D. pseudospathaceum*	*Blicca bjoerkna*	S	BBD6	H8	KY653998	KY654052
*D. pseudospathaceum*	*Blicca bjoerkna*	S	BBD7	H10	KY653999	
*D. pseudospathaceum*	*Blicca bjoerkna*	S	BBD8	H11	KY654000	
*D. pseudospathaceum*	*Blicca bjoerkna*	H	BBD9	H4	KY654001	
*D. pseudospathaceum*	*Blicca bjoerkna*	S	BBD10	H9	KY654002	
*D. pseudospathaceum*	*Cyprinus carpio*	S	CCD	H1	KY654003	KY654053
*D. pseudospathaceum*	*Gymnocephalus schraetser*	H	GSD	H4	KY654004	
*D. pseudospathaceum*	*Leuciscus aspius*	S	LAD3	H13	KY654005	
*D. pseudospathaceum*	*Leuciscus aspius*	S	LAD4	H1	KY654006	
*D. pseudospathaceum*	*Leuciscus aspius*	S	LAD5	H2	KY654007	
*D. pseudospathaceum*	*Leuciscus aspius*	S	LAD6	H6	KY654008	
*D. pseudospathaceum*	*Leuciscus aspius*	S	LAD7	H5	KY654009	KY654054
*D. pseudospathaceum*	*Leuciscus aspius*	S	LAD8	H5	KY654010	
*D. pseudospathaceum*	*Leuciscus aspius*	H	LAD9	H4	KY654011	
*D. pseudospathaceum*	*Leuciscus idus*	S	LID1	H1	KY654012	KY654055
*D. pseudospathaceum*	*Leuciscus idus*	S	LID2	H12	KY654013	
*D. pseudospathaceum*	*Lota lota*	H	LLD	H3	KY654014	
*D. pseudospathaceum*	*Vimba vimba*	S	VVD3	H1	KY654015	KY654056
*D. pseudospathaceum*	*Vimba vimba*	H	VVD4	H1	KY654016	
‘*D. mergi* Lineage 2’	*Abramis brama*	S	ABD15	H2	KY654017	
‘*D. mergi* Lineage 2’	*Abramis brama*	S	ABD16	H4	KY654018	KY654057
‘*D. mergi* Lineage 2’	*Abramis brama*	S	ABD17	H1	KY654019	KY654058
‘*D. mergi* Lineage 2’	*Abramis brama*	S	ABD18	H2	KY654020	KY654059
‘*D. mergi* Lineage 2’	*Alburnus alburnus*	H	AAD1	H2	KY654021	
‘*D. mergi* Lineage 2’	*Alburnus alburnus*	S	AAD2	H5	KY654022	KY654060
‘*D. mergi* Lineage 2’	*Alburnus alburnus*	H	AAD3	H1	KY654023	KY654061
‘*D. mergi* Lineage 2’	*Alburnus alburnus*	H	AAD4	H1	KY654024	
‘*D. mergi* Lineage 2’	*Alburnus alburnus*	H	AAD5	H1	KY654025	
‘*D. mergi* Lineage 2’	*Alburnus alburnus*	H	AAD6	H1	KY654026	
‘*D. mergi* Lineage 2’	*Ballerus sapa*	H	BSD5	H7	KY654027	KY654062
‘*D. mergi* Lineage 2’	*Blicca bjoerkna*	S	BBD11	H3	KY654028	KY654063
‘*D. mergi* Lineage 2’	*Blicca bjoerkna*	S	BBD12	H1	KY654029	KY654064
‘*D. mergi* Lineage 2’	*Blicca bjoerkna*	H	BBD13	H1	KY654030	
‘*D. mergi* Lineage 2’	*Chondrostoma nasus*	S	CND3	H1	KY654031	KY654065
‘*D. mergi* Lineage 2’	*Vimba vimba*	H	VVD5	H6	KY654032	
‘*D. mergi* Lineage 2’	*Vimba vimba*	H	VVD6	H1	KY654033	KY654066
*Diplostomum* sp. A	*Blicca bjoerkna*	S	BBD14	–	KY654034	
*Diplostomum* sp. B	*Carassius gibelio*	S	CGD	–	KY654035	
*Diplostomum* sp. C	*Rutilus rutilus*	S	RRD3	–	KY654036	

*Abbreviations*: *H* Hungary, *S* Slovakia

The intraspecific divergence (uncorrected p-distance range), observed within the newly generated *cox*1 sequences, ranged between 0 and 1.71% (mean 0.56%) for *D*. *pseudospathaceum*, 0–1.95% (mean 0.82%) for *D*. *spathaceum* and 0–1.71% (mean 0.47%) for ‘*D. mergi* Lineage 2’. The three singletons exhibited high levels of divergence compared with the isolates of *Diplostomum* spp. included in the analyses: 7.1–15.6% for *Diplostomum* sp. A; 5.6–15.9% for *Diplostomum* sp. B; and 11.5–15.0% for *Diplostomum* sp. C.

The newly generated sequences for the three *Diplostomum* spp. were collapsed into 16 haplotypes for *D. spathaceum*, 15 haplotypes for *D*. *pseudospathaceum* and 7 haplotypes for ‘*D. mergi* Lineage 2’. Of these, *D. spathaceum* and *D. pseudospathaceum* had 7 unique haplotypes each (H1, H8, H9, H11, H14, H15, H16 and H3, H6, H8, H9, H11, H13, H14, respectively); and ‘*D. mergi* Lineage 2’ had 4 unique haplotypes (H3, H4, H5, H6).

Nine haplotypes of *D. spathaceum* were shared among isolates studied here and previously published sequences, predominantly generated in studies carried out in Europe (Germany, Iceland and Spain; see Georgieva et al. [7]; Pérez-del-Olmo et al. [3]; Selbach et al. [10]) (see Table 3 for details). Notably, four haplotypes (H2, H5, H6 and H10) were shared between isolates from all three hosts in the species life-cycle (first intermediate hosts: *Radix auricularia* (L.) and *Radix peregra* (Müller); definitive hosts: *Larus argentatus* (*s.l.*) and *L. ridibundus*; second intermediate host: a number of fish species). Due to the geographical coverage of the previous studies, most of the shared haplotypes originate from Europe; however, sequence matches for isolates from Asia [6] indicate a wider distribution of six haplotypes (Iraq: H2, H5, H7 and H10; China: H2, H13) (Table 3). It is also worth noting that four of the haplotypes were shared with haplotypes implicated in a case of diplostomiasis in aquaculture of *Pseudochondrostoma willkommii* (Steindachner) [3].

**Table 3:** Details for the hosts, localities and GenBank accession numbers for the shared haplotypes of *Diplostomum* spp. identified in fishes from the River Danube

Species/Haplotype	Present study		Published isolates with matching sequences			
	Isolate code^a^	Host	GenBank ID	Host	Origin	Reference
*Diplostomum spathaceum*						
H2	ABD8; LAD2; RPD2	*A. brama*; *L. aspius*; *R. pigus*	JX986889; KR149550; KR149553; JX986888; KJ726433, KJ726434; KR271463; KR271451; KR271426; KR271430; JX986887	Snails: *Radix auricularia* Fishes: *Abramis brama*; *Acanthobrama marmid*; *Barbus luteus*; *Cyprinion macrostomum*; *Gasterosteus aculeatus* Birds: *Larus cachinnans*	China; Czech Republic; Germany; Iceland; Iraq	[6, 7, 9, 10]
H3	ABD9; RPD4; SGD	*A. brama*; *R. pigus*; *S. glanis*	JX986894; KR271417	Fishes: *Gasterosteus aculeatus*; *Perca fluviatilis*	Germany; Italy	[6, 7]
H4	ARD; BBD2	*A. ruthenus*; *B. bjoerkna*	JX986893; KP025775; KP025785; KJ726438; KR271462	Fishes: *Gasterosteus aculeatus*; *Pseudochondrostoma willkommii*; *Salvelinus alpinus*; *Silurus glanis* Birds: *Larus ridibundus*	Germany; Iceland; Romania; Spain	[3, 6, 7, 9]
H5	ABD4; RPD1	*A. brama*; *R. pigus*	JX986892; KR149551; KR271422, KR271429; KP025783; KP025772	Snails: *Radix auricularia.* Fishes: *Cyprinion macrostomum*; *Pseudochondrostoma willkommii* Birds: *Larus argentatus*; *L. argentatus michahellis*	Germany; Iraq; Poland; Spain	[3, 6, 7, 10]
H6	BBD1	*B. bjoerkna*	KR149547, KR149548; KP025781; KP025778; KP025774; KJ726435, KJ726436; KR271431	Snails: *Radix auricularia*; *Radix peregra* Fishes: *Gasterosteus aculeatus*; *Misgurnus anguillicaudatus*; *Pseudochondrostoma willkommii* Birds: *Larus argentatus michahellis*	Germany; Iceland; Spain	[3, 6, 9, 10]
H7	CND1	*C. nasus*	JX986891; KR149552; JX986890; KP025786, KP025782; KR271452; KR271423	Snails: *Radix auricularia* Fishes: *Acanthobrama marmid*; *Cyprinion macrostomum*; *Gasterosteus aculeatus*; *Pseudochondrostoma willkommii*	Germany; Iraq; Spain	[3, 6, 7, 10]
H10	ABD7	*A. brama*	KR149549; KP025779; KR271428; JX986895	Snails: *Radix auricularia* Fishes: *Barbus luteus*; *Misgurnus anguillicaudatus* Birds: *Larus cachinnans*	Germany; Iraq; Poland; Spain	[3, 6, 7, 10]
H12	ABD6	*A. brama*	KR271420	Fishes: *Perca fluviatilis*	Italy	[6]
H13	LAD1	*L. aspius*	KR271459	Fishes: *Abramis brama*	China	[6]
*Diplostomum pseudospathaceum*						
H1	ABD10; ABD11; BBD4; BSD1; CCD; LAD4; LID1; VVD3; VVD4	*A. brama*; *B. bjoerkna*; *B. sapa*; *C. carpio*; *L. aspius*; *L. idus*; *V. vimba*	JX986899; JX986900; KR149529; KR149535; KR149536; KR271088; JX986901; KR271090; KR271091	Snails: *Lymnaea stagnalis*; *Stagnicola palustris* Fishes: *Silurus glanis*	Germany; Romania	[6, 7, 10]
H2	ABD12; BSD4; LAD5	*A. brama*; *B. sapa*; *L. aspius*	JX986897; KR149534; KR149533; KR149532; KR149530; JX986898; KR149541; KR271093; JX986896	Snails: *Lymnaea stagnalis*; *Stagnicola palustris* Fishes: *Cyprinus carpio* Birds: *Larus cachinnans*	Czech Republic; Germany; Romania	[6, 7, 10]
H4	BBD9; GSD; LAD9	*B. bjoerkna*; *G. schraetsor*; *L. aspius*	KR149546	Snails: *Stagnicola palustris*	Germany	[10]
H5	LAD7; LAD8	*L. aspius*	JX986902; JX986903	Fishes: *Gasterosteus aculeatus*	Germany	[7]
H7	BBD5	*B. bjoerkna*	KR149542	Snails: *Stagnicola palustris*	Germany	[10]
H10	BBD7	*B. bjoerkna*	JX986907	Snails: *Lymnaea stagnalis*	Germany	[7]
H12	LID2	*L. idus*	KR149531	Snails: *Lymnaea stagnalis*	Germany	[10]
H15	ABD14	*A. brama*	KR149537	Snails: *Stagnicola palustris*	Germany	[10]
‘*Diplostomum mergi* Lineage 2’						
H1	AAD3; AAD4; AAD5; AAD6; ABD17; BBD12; BBD13; CND3; VVD6	*A. alburnus*; *A. brama*; *B. bjoerkna*; *C. nasus*; *V. vimba*	JX986874; JX986875; JX986876; KR149522; KR149521; KR149520; KR149518; KR149517; KR149515; KR149514	Snails: *Radix auricularia*	Germany	[7, 10]
H2	AAD1; ABD15; ABD18	*A. alburnus*; *A. brama*	KR149523; KR149519; KR149516	Snails: *Radix auricularia*	Germany	[10]
H7	BSD5	*B. sapa*	KR271082	Fishes: *Abramis brama*	China	[6]

^a^See Table 2 for details

Of the 15 haplotypes of *D. pseudospathaceum*, 8 were shared with previously reported isolates, predominantly from the first intermediate hosts, *Lymnaea stagnalis* (L.) and *Stagnicola palustris* (Müller), from the Czech Republic, Germany and Romania [6, 7, 10]; among these, a single haplotype (H2) was shared between isolates from all three hosts in the species life-cycle (Table 3). Finally, three haplotypes of ‘*D. mergi* Lineage 2’ were shared with isolates from the snail host *R. auricularia* in Germany (H1 and H2) and one with a metacercaria from *A. brama* in China (H7, see Table 3).

**Fig. 3:**
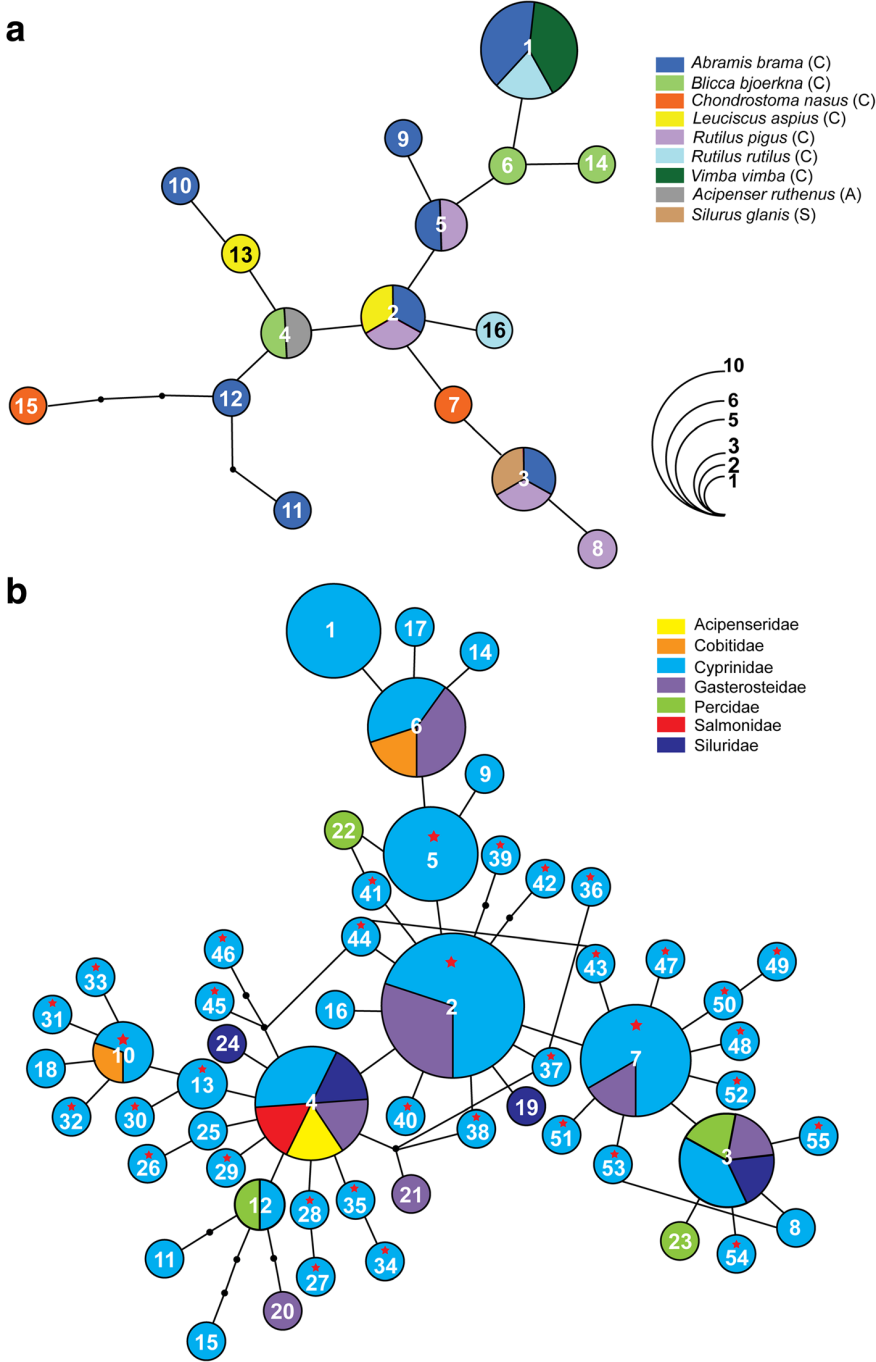
Haplotype networks for *Diplostomum spathaceum*: (**a**) based on the novel *cox*1 sequences from metacercarial isolates sampled from nine fish species in the River Danube; (**b**) based on all currently published *cox*1 sequences from metacercarial isolates sampled from fishes in Europe and Asia. Numbers indicate the haplotype code number (see Table 2 and Additional file 2: Table S2 for details). Black dots represent inferred unsampled intermediate haplotypes and connective lines represent one mutational step. Pie chart size is proportional to the number of isolates sharing a haplotype; haplotype frequency is indicated by colourless semicircles. Hosts reported in this study (**a**) and host families (**b**) are colour-indicated; stars indicate haplotypes recovered in Asia. *Abbreviations*: A, Acipenseridae; C, Cyprinidae; S, Siluridae

**Fig. 4:**
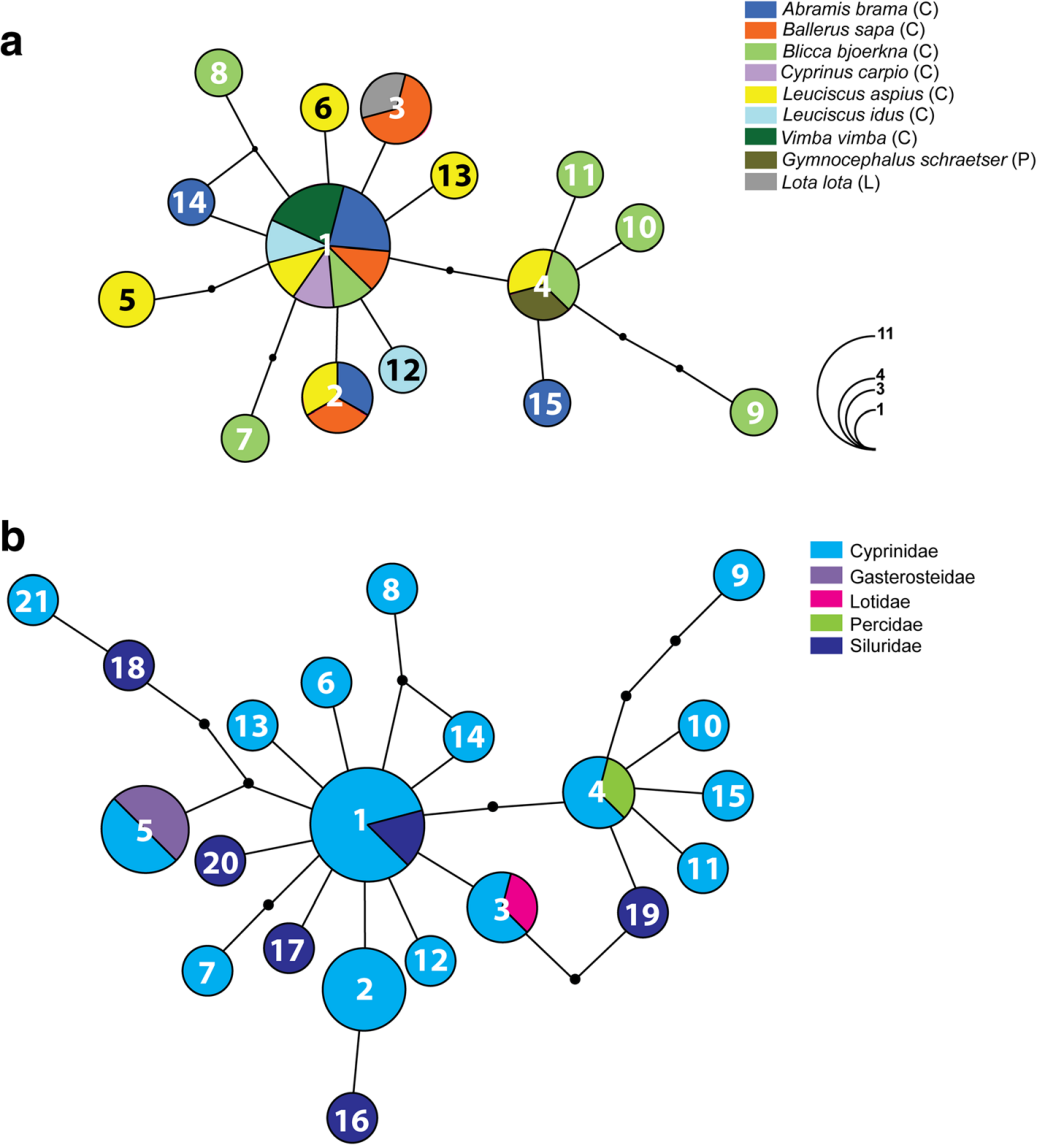
Haplotype networks for *Diplostomum pseudospathaceum*: (**a**) based on the novel *cox*1 sequences from metacercarial isolates sampled from nine fish species in the River Danube; (**b**) based on all currently published *cox*1 sequences from metacercarial isolates sampled from fishes in Europe. Numbers indicate the haplotype code number (see Table 2 and Additional file 2: Table S2 for details). Black dots represent inferred unsampled intermediate haplotypes and connective lines represent one mutational step. Pie chart size is proportional to the number of isolates sharing a haplotype; haplotype frequency is indicated by colourless semicircles. Hosts reported in this study (**a**) and host families (**b**) are colour-indicated. *Abbreviations*: C, Cyprinidae; L, Lotidae; P, Percidae

The *cox*1 haplotype networks for *D. spathaceum* and *D. pseudospathaceum,* generated by statistical parsimony analysis, are presented in Figs. 3 and 4, respectively. For both species, haplotypes identified in the present material were sampled from 9 fish host species and there was no indication of genetic structuring associated with the host. The ancestral haplotype (H1) of *D. spathaceum* was recovered as unique and represented by isolates from 3 cyprinid hosts (*A. brama*, *R. rutilus* and *V. vimba*). Two other haplotypes (H2 and H3) were shared by isolates from 3 fish hosts each (*A. brama*, *L. aspius* and *R. pigus* and *A. brama*, *R. pigus* and *S. glanis*, respectively) (Fig. 3a). The cyprinid *A. brama* was the host with the largest haplotype diversity (8 haplotypes; 2 unique).

Figure 3b illustrates a haplotype network including all available sequence data for *D. spathaceum* from fish hosts in Europe and Asia. A total of 68 sequences was added for isolates from 12 fish species of five families: Cyprinidae (7 species; Locke et al. [6], Pérez-del-Olmo et al. [3]); Gasterosteidae (1 species; Georgieva et al. [7], Blasco-Costa et al. [9]); Cobitidae (1 species; Pérez-del-Olmo et al. [3]); Percidae (1 species; Locke et al. [6]); Salmonidae (1 species; Blasco-Costa et al. [9]) and Siluridae (1 species; Locke et al. [6]) (see Additional file 2: Table S2 for details). This expanded dataset comprising 94 sequences (trimmed to 402 nt) for isolates from 17 fish host species of 7 families revealed a much higher haplotype diversity (55 haplotypes) and a generally similar pattern for the most common haplotypes. However, a large number of haplotypes were represented by singletons (45 haplotypes: H8, H9, H11, H14-H55, see Additional file 2: Table S2) and H2 was the most common haplotype in the expanded network. A total of 30 haplotypes was identified in isolates sampled recently in China (*n* = 4) and Iraq (*n* = 26) by Locke et al. [6], and five haplotypes (H2, H5, H7, H10 and H13) were shared by isolates from Europe and Asia (Fig. 3b; Table 3). Notably, three of the five major haplotypes (H2-H4) recovered from different host species in the River Danube (Fig. 3a) also exhibited low host-specificity at the level of host family (associated with fish hosts of 2–5 families, see Fig. 3b) whereas haplotypes H1 and H5 appear to be restricted to the Cyprinidae based on the currently available data.

*Diplostomum pseudospathaceum* exhibited a marked contrast in haplotype network structure (star-shaped network, indicative of range expansion, see Fig. 4a) compared to the more complex network for *D. spathaceum*. The ancestral haplotype (H1) was shared among isolates from 7 of the 9 fish hosts (all cyprinids). The largest haplotype diversity was also found in cyprinid fishes: *B*. *bjoerkna* (7 haplotypes; 3 unique) followed by *L. aspius* (6 haplotypes, 2 unique). The haplotype network, including all available sequence data for *D. pseudospathaceum* from fish hosts in Europe (Fig. 4b) (12 host species of 5 families), includes 11 additional sequences for isolates from 3 fish species of 3 families: Cyprinidae (2 species; Locke et al. [6]); Gasterosteidae (1 species; Georgieva et al. [7]); and Siluridae (1 species; Locke et al. [6]) (see Additional file 2: Table S2 for details). This resulted in adding 6 new haplotypes (all singletons) to the dataset (41 sequences, trimmed to 402 nt; 21 haplotypes, see Additional file 2: Table S2). The haplotype network (Fig. 4b) closely resembled that for fishes sampled in the River Danube (Fig. 4a). Three of the four haplotypes identified in isolates from different fish species in the River Danube were also recovered in non-cyprinid fishes (Fig. 4b) (H1: Siluridae; H3: Lotidae; and H4: Percidae) and one haplotype (H5) was also identified in isolates from *G. aculeatus* (Gasterosteidae) (Georgieva et al. [7]).

**Fig. 5:**
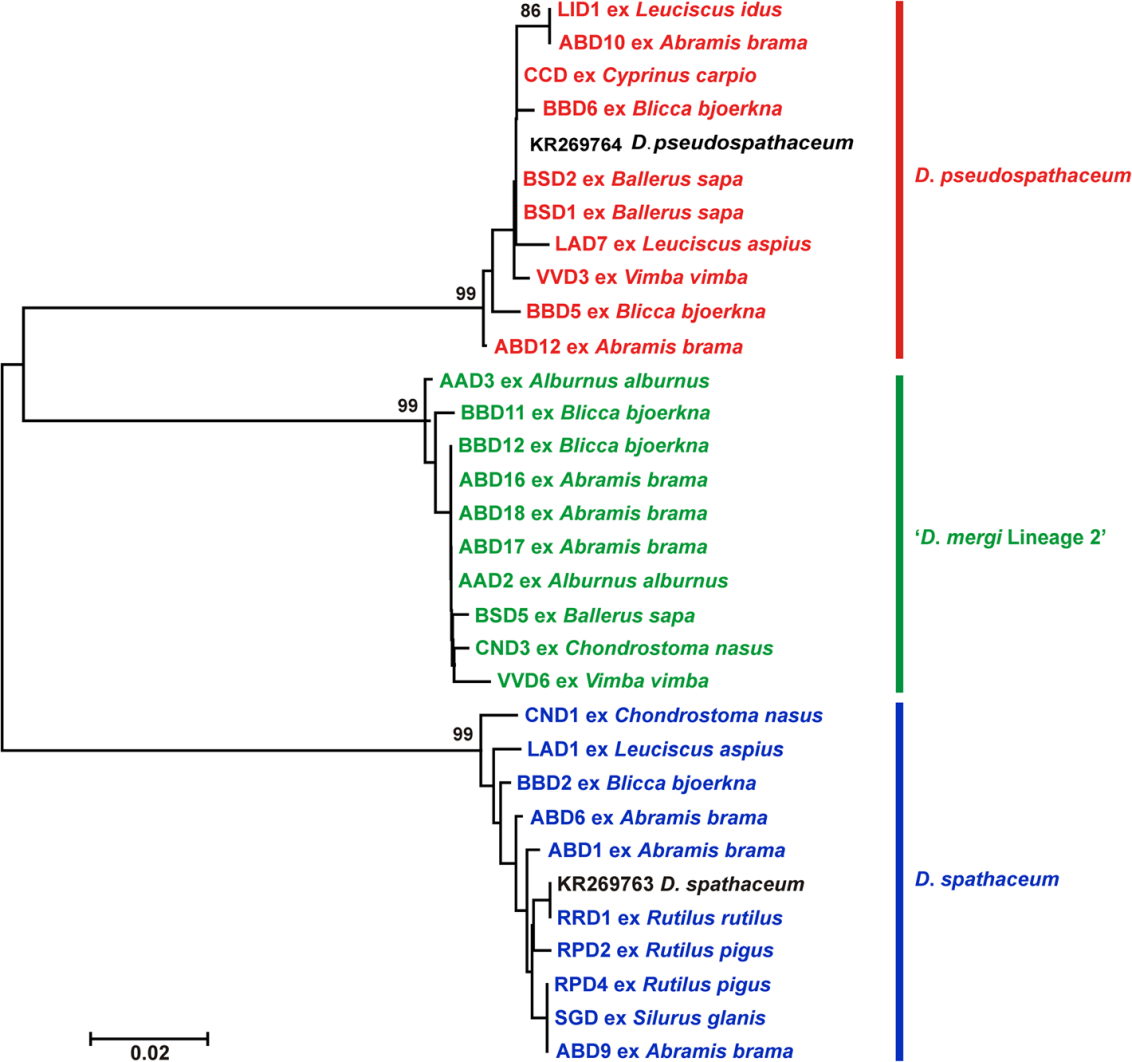
Neighbour-joining (NJ) phylogram for *Diplostomum* spp. reconstructed using 30 newly generated and two *nad*3 sequences retrieved from GenBank. The scale-bar indicates the expected number of substitutions per site. Codes for the newly sequenced isolates are provided in Table 2

To aid further exploration of species boundaries among the most widespread lens-infecting *Diplostomum* spp., the *nad*3 gene was selected based on its lower level of sequence conservation (83.3%) compared with the ‘barcode’ region of the *cox*1 gene (90.6%) (see Brabec et al. [25]). A total of 30 complete *nad*3 sequences (357 nt) were generated for the three species identified based on the *cox*1 gene subsampling (10 isolates per species; see Table 2 for details). NJ analysis of the *nad*3 dataset depicted three distinct well-supported monophyletic clades corresponding to the *cox*1 lineages (Fig. 5). The levels of the interspecific divergence for the *nad*3 gene was distinctly higher with minimum p-distance values well above the maximum values for *cox*1 (14.6–15.7 *vs* 9–11.2%) (Table 4). It is worth noting that the use of the newly designed primers resulted in successful amplification of *nad*3 in the distantly related lineage of the “*D. mergi*” complex of cryptic species.

**Table 4:** Levels of divergence (p-distance in %) for *cox*1 and *nad*3 gene sequences in interspecific comparisons of *Diplostomum* spp.

Species comparison	*cox*1	*nad*3
*D. pseudospathaceum* *vs* *D. spathaceum*	9.0–10.7	15.7–17.4
*D. spathaceum* *vs* ‘*D*. *mergi* Lineage 2’	10.0–11.7	15.4–16.8
*D. pseudospathaceum* *vs* ‘*D*. *mergi* Lineage 2’	11.2–12.9	14.6–16.2

### Descriptions of the molecular voucher material

Comparisons based on live metacercariae of the most frequent species in this study, *D*. *spathaceum*, *D*. *pseudospathaceum* and ‘*D. mergi* Lineage 2’ revealed that metacercariae of *D*. *spathaceum* exhibit the highest mean values for the width of the body, the length of the hindbody, and the size of the oral sucker, pseudosuckers and pharynx. Live metacercariae of *D*. *pseudospathaceum* were characterised by the lowest mean values for the size of the body, pseudosuckers and holdfast organ whereas those of ‘*D. mergi* Lineage 2’ exhibited the highest mean values for the length of the body and the size of the ventral sucker and holdfast organ. Surprisingly, fixed metacercariae of ‘*D. mergi* Lineage 2’ demonstrated the highest mean values for the size of the body, pseudosuckers, ventral sucker, holdfast organ and hindbody whereas the dimensions of specimens of *D. spathaceum* and *D*. *pseudospathaceum* were rather similar (see Tables 5, 6). We have therefore provided morphological and morphometric characterisation based on both live and fixed material.

**Table 5:** Comparative metrical data for metacercariae of *Diplostomum spathaceum*

Host Source	Multiple hosts^a^ Present study		*Gasterosteus aculeatus* L.; *Salvelinus alpinus* (L.) Faltýnková et al. [16]				*Cyprinus carpio* L. Pérez-del-Olmo et al. [3]	
	Fixed		Live		Fixed		Fixed	
Variable	Range (*n* = 21)	Mean	Range	Mean	Range	Mean	Range	Mean
BL	288–415	346	360–570	498	262–574	376	277–453	376
BW	241–333	288	252–332	286	171–313	235	198–295	248
HL	17	17	36–80	53	22–67	41	10–26	16
PSL	46–61	53	–	–	35–40	37	44–55	48
PSW	24–36	29	–	–	–	–	22–30	26
OSL	40–54	47	44–65	57	44–64	52	40–57	45
OSW	37–52	46	44–72	60	41–72	50	36–41	39
PHL	30–42	38	36–51	42	29–45	35	29–43	37
PHW	16–26	21	20–32	26	16–19	17	19–26	23
VSL	38–51	45	35–55	45	40–56	49	30–43	38
VSW	48–61	54	38–62	50	34–53	43	33–48	43
AVS	135–248	181	–	–	–	–	–	–
HOL	67–99	84	78–131	104	72–82	77	63–89	75
HOW	92–130	112	83–181	131	63–95	81	59–90	80

*Abbreviations*: *BL* body length, *BW* body width, *HL* hindbody length, *PSL* pseudosucker length, *PSW* pseudosucker width, *OSL* oral sucker length, *OSW* oral sucker width, *PHL* pharynx length, *PHW* pharynx width, *VSL* ventral sucker length, *VSW* ventral sucker width, *AVS* distance from anterior extremity of body to ventral sucker, *HOL* holdfast organ length, *HOW* holdfast organ width

^a^ *Acipenser ruthenus* L.; *Abramis brama* (L.); *Blicca bjoerkna* (L.); *Chondrostoma nasus* (L.); *Leuciscus aspius* (L.); *Rutilus pigus* (Lacépède); *Rutilus rutilus* (L.); *Vimba vimba* (L.); *Silurus glanis* L.

**Table 6:** Comparative metrical data for metacercariae of *Diplostomum* spp.

Species	*Diplostomum pseudospathaceum*		*Diplostomum pseudospathaceum*		‘*Diplostomum mergi* Lineage 2’		*Diplostomum* sp. A	*Diplostomum* sp. B	*Diplostomum* sp. C
Host	Multiple hosts^a^		*Cyprinus carpio* L.		Multiple hosts^b^		*Blicca bjoerkna* (L.)	*Carassius gibelio* (Bloch)	*Rutilus* *rutilus* (L.)
Source	Present study		Niewiadomska [26]		Present study		Present study	Present study	Present study
	Fixed		Fixed		Fixed		Fixed	Fixed	Fixed
Variable	Range (*n* = 24)	Mean	Range	Mean	Range (*n* = 18)	Mean	*n* = 1	*n* = 1	*n* = 1
BL	288–447	364	347–458	381	362–485	420	338	426	381
BW	234–301	264	162–296	201	242–338	287	242	304	278
HL	19–19	19	–	–	14–45	26	20	19	16
PSL	40–65	52	–	–	52–68	60	47–52	56–58	61–67
PSW	25–35	30	–	–	31–36	34	–	–	–
OSL	39–56	47	42–52	45.8	41–53	47	37	46	47
OSW	36–53	44	30–51	37.7	34–49	43	44	41	47
PHL	32–45	38	28–35	31.8	30–45	38	30	41	30
PHW	19–25	21	17–30	20.4	19–23	22	20	22	–
VSL	33–53	42	34–42	38.9	40–62	51	51	51	43
VSW	43–56	51	35–51	42.2	49–70	61	64	59	49
AVS	158–243	191	–	–	174–261	208	143	215	174
HOL	68–96	82	62–81	67.5	95–115	104	65	115	–
HOW	79–126	99	54–76	61.7	102–187	136	106	136	–

*Abbreviations*: *BL* body length, *BW* body width, *HL* hindbody length, *PSL* pseudosucker length, *PSW* pseudosucker width, *OSL* oral sucker length, *OSW* oral sucker width, *PHL* pharynx length, *PHW* pharynx width, *VSL* ventral sucker length, *VSW* ventral sucker width, *AVS* distance from anterior extremity of body to ventral sucker, *HOL* holdfast organ length, *HOW* holdfast organ width

^a^ *Abramis brama* (L.); *Ballerus sapa* (Pallas); *Blicca bjoerkna* (L.); *Cyprinus carpio* L.; *Leuciscus aspius* (L.); *L*. *idus* (L.); *Vimba vimba* (L.); *Lota lota* (L.); *Gymnocephalus schraetser* (L.)

^b^ *Abramis brama* (L.); *Alburnus alburnus* (L.); *Ballerus sapa* (Pallas); *Blicca bjoerkna* (L.); *Chondrostoma nasus* (L.); *Vimba vimba* (L.)

**Fig. 6:**
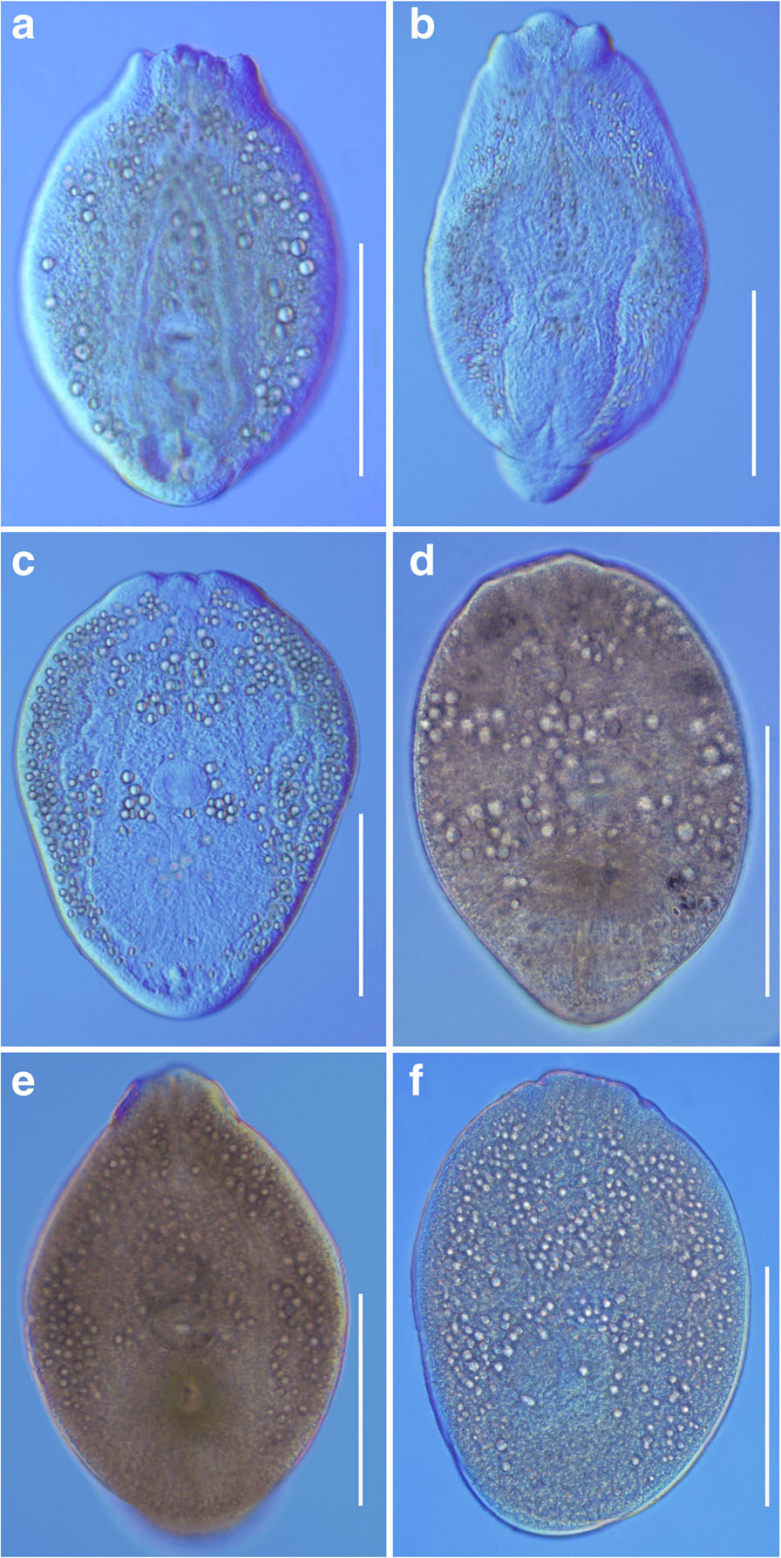
Metacercariae of *Diplostomum* spp. (**a**-**c**, live; **d**-**f**, fixed). **a** *D*. *spathaceum* from the eye lens of *Rutilus pigus* (hologenophore; GenBank KY653979 and KY654043). **b** *D*. *pseudospathaceum* from the eye lens of *Abramis brama* (hologenophore; GenBank KY653989 and KY654048). **c** ‘*D. mergi* Lineage 2’ from the eye lens of *Abramis brama* (hologenophore; GenBank KY654020 and KY654059). **d** *Diplostomum* sp. A from the eye lens of *Blicca bjoerkna* (hologenophore; GenBank KY654034). **e** *Diplostomum* sp. B from the eye lens of *Carassius gibelio* (hologenophore; GenBank KY654035). **f** *Diplostomum* sp. C from the eye lens of *Rutilus rutilus* (hologenophore; GenBank KY654036). *Scale-bars*: 200 μm

Unfortunately, the single metacercariae of *Diplostomum* sp. A, *Diplostomum* sp. B and *Diplostomum* sp. C were fixed in the field and their descriptions are based on fixed material. Nevertheless, comparisons based on fixed metacercariae of the six forms recovered in the present study indicate that the sucker ratios and the number and relative size of the excretory concretions are the most prominent characters that can be used for their discrimination. *Diplostomum* sp. A and B exhibited the largest values for the sucker width ratio and were characterised by having large excretory concretions, similar to those observed in *D. spathaceum*. However, the metacercaria of *Diplostomum* sp. B is much larger (426 × 304 *vs* a mean of 346 × 288 μm for *D. spathaceum*) and the excretory concretions in the metacercaria of *Diplostomum* sp. A also appear larger than in the metacercaria of *D. spathaceum* (Fig. 6). The metacercaria of *Diplostomum* sp. C can be distinguished from the other five forms in having the largest number of excretory concretions (482 *vs* a maximum of 254, 360, 440 in *D. spathaceum*, *D. pseudospathaceum* and ‘*Diplostomum mergi* Lineage 2’, respectively, and 154 and 261 in *Diplostomum* sp. A and *Diplostomum* sp. B, respectively) (see also Fig. 6).

### *Diplostomum spathaceum* (Rudolphi, 1819)

***Hosts*** **:** *Acipenser ruthenus* L. (Chondrostei: Acipenseridae), *Abramis brama* (L.), *Blicca bjoerkna* (L.), *Chondrostoma nasus* (L.), *Leuciscus aspius* (L.), *Rutilus pigus* (Lacépède), *Rutilus rutilus* (L.), *Vimba vimba* (L.) (Teleostei: Cyprinidae); *Silurus glanis* L. (Teleostei: Siluridae).

***Prevalence*** **:** *A. ruthenus*: 1/1 (Slovakia, S); *A. brama*: 75% (29/40, S); *B. bjoerkna*: 1/5 (Hungary, H), 1/8 (S); *C. nasus*: 2/7 (H), 1/5 (S); *L. aspius*: 3/6 (H), 1/4 (S); *R. pigus*: 2/3 (S); *R. rutilus*: 1/1 (H), 2/8 (S); *V. vimba*: 2/4 (S); *S. glanis*: 1/1 (S).

***Representative DNA sequences*** **:** KY653961–KY653986 (*cox*1); KY654037–KY654046 (*nad*3).

### Description

[Based on 20 live metacercariae. Metrical data for fixed material are provided in Table 5; Fig. 6a.] Body oval, 349–601 × 265–442 (474 × 341), with maximum width just anterior to ventral sucker. Oral sucker elongate-oval, 51–80 × 46–69 (62 × 57). Pseudosuckers strongly muscular, elongate-oval, 58–90 × 31–51 (76 × 41). Oral opening terminal; prepharynx absent; pharynx elongate-oval, 32–47 × 20–39 (40 × 28); oesophagus short, bifurcates close posterior to pharynx; caeca long, narrow, reach posterior to holdfast organ. Ventral sucker transversely oval, 34–64 × 38–66 (50 × 56), smaller or equal to oral sucker (sucker width ratio 1:0.83–1.19 (1:1.01), posterior to mid-body length. Distance from anterior extremity of body to ventral sucker 191–365 (262). Holdfast organ relatively small, transversely oval, bipartite, contiguous with ventral sucker, 71–153 × 78–180 (108 × 124). Excretory vesicle small, V-shaped; reserve excretory system of diplostomid type; excretory concretions relatively large, 171–346 (246) in number, grouped into 2 lateral extracaecal [106–254 (179) excretory concretions] and 1 median [39–109 (67) excretory concretions] fields. Hindbody 34–59 (44) long.

### Remarks

The morphology of the present metacercariae of *D*. *spathaceum* (Fig. 6a) agrees with the descriptions of metacercariae of *D*. *spathaceum* by Faltýnková et al. [16] and Pérez-del-Olmo et al. [3] with some variations. The present live specimens differ from the live material described by Faltýnková et al. [16] by having on average shorter and wider body, somewhat larger pseudosuckers and ventral sucker, narrower holdfast organ and a different sucker width ratio (mean 1:1.01 *vs* 1:0.84) (also see Table 5). Similarly, the present fixed specimens differ from the fixed material described by Faltýnková et al. [16] and Pérez-del-Olmo et al. [3] in having on average shorter and wider body and larger pseudosuckers and ventral sucker and a distinctly wider holdfast organ. The number of excretory concretions in *D. spathaceum* falls within the range provided by Shigin [1] but the mean is distinctly higher: 171–346 (246) *vs* 117–401 (143).

Our study adds 8 fish species to the hosts of *D. spathaceum* in Europe confirmed by molecular evidence. Previous records include *Gasterosteus aculeatus* L. in Germany [7]; *G. aculetaus* and *Salvelinus alpinus* (L.) in Iceland [9]; *Misgurnus anguillicaudatus* (Cantor), *S. glanis* and *P. willkommii* in Spain [3]; and *Perca fluviatilis* L. in Italy and *S. glanis* in Romania [6]. Among these hosts, cyprinids predominate (7 species) and are more diverse; a very high prevalence (75%) was also registered in a cyprinid (*A. brama*; present study).

### *Diplostomum pseudospathaceum* Niewiadomska, 1984

***Hosts*** **:** *Abramis brama* (L.), *Ballerus sapa* (Pallas), *Blicca bjoerkna* (L.), *Cyprinus carpio* L., *Leuciscus aspius* (L.), *L*. *idus* (L.), *Vimba vimba* (L.) (Teleostei: Cyprinidae); *Lota lota* (L.) (Teleostei: Lotidae), *Gymnocephalus schraetser* (L.) (Teleostei: Percidae).

***Prevalence*** **:** *A. brama*: 50% (20/40, S); *B. sapa*: 1/1 (S); *B. bjoerkna*: 3/5 (H), 5/8 (S); *C. carpio*: 1/3 (S); *L. aspius*: 2/5 (H), 3/4 (S); *L*. *idus*: 1/1 (S); *V. vimba*: 1/5 (H), 1/4 (S); *L. lota*: 1/2 (H); *G. schraetser*: 1/5 (H).

***Representative DNA sequences*** **:** KY653987–KY654016 (*cox*1); KY654047–KY654056 (*nad*3).

### Description

[Based on 18 live metacercariae. Metrical data for fixed material are provided in Table 6; Fig. 6b.] Body elongate-oval, 325–490 × 234–410 (406 × 306), with maximum width just anterior to ventral sucker. Oral sucker elongate-oval, 48–65 × 43–58 (55 × 50). Pseudosuckers strongly muscular, elongate-oval, 42–73 × 26–43 (54 × 33). Oral opening terminal; prepharynx short or absent; pharynx elongate-oval, 31–52 × 19–37 (38 × 24); oesophagus short, bifurcates close posterior to pharynx; caeca long, narrow, reach posterior to holdfast organ. Ventral sucker transversely oval, 37–56 × 45–66 (47 × 55), smaller or larger than oral sucker [sucker width ratio 1:0.93–1.35 (1:1.11)], slightly posterior to mid-body length. Distance from anterior extremity of body to ventral sucker 177–279 (216). Holdfast organ relatively small, transversely oval, bipartite, contiguous with ventral sucker, 69–111 × 88–170 (90 × 115). Excretory vesicle small, V-shaped; reserve excretory system of diplostomid type; excretory concretions small, 185–360 (241) in number, grouped into 2 lateral extracaecal [122–244 (164) excretory concretions] and 1 median [57–116 (77) excretory concretions] fields. Hindbody 19–47 (31) long.

### Remarks

The present metacercariae were identified as *D*. *pseudospathaceum* based on molecular data. The metrical data for the present material (fixed specimens) exhibit overlapping ranges with the data for experimentally developed metacercariae of *D*. *pseudospathaceum* described by Niewiadomska [26] but differ in the possesion of on average shorter and wider body, wider suckers and distinctly wider holdfast organ (Table 6). Shigin [1] reported 151–309 (234) excretory concretions for *D. pseudospathaceum* (as *D. chromatophorum*); these values agree very well with our observations, i.e. 185–360 (241).

Our study reports nine fish hosts for *D. pseudospathaceum* in Europe confrmed by sequencing. Previous molecularly identified records in fishes are few: *G. aculeatus* in Germany [7] and *C. carpio* and *S. glanis* in Romania [6]. Among the hosts studied here, cyprinids predominated (7 species) with a high prevalence in *A. brama* (50%).

### ‘*Diplostomum mergi* Lineage 2’ *sensu* Georgieva et al. (2013)

***Hosts*** **:** *Abramis brama* (L.), *Alburnus alburnus* (L.), *Ballerus sapa* (Pallas), *Blicca bjoerkna* (L.), *Chondrostoma nasus* (L.), *Vimba vimba* (L.) (Teleostei: Cyprinidae).

***Prevalence*** **:** *A. brama*: 58% (23/40, S); *A. alburnus*: 3/5 (H), 1/3 (S); *B. sapa*: 1/8 (H), 1/1 (S); *B. bjoerkna*: 1/5 (H), 2/8 (S); *C. nasus*: 1/4 (S); *V. vimba*: 4/5 (H).

***Representative DNA sequences*** **:** KY654017–KY654033 (*cox*1); KY654057–KY654066 (*nad*3).

### Description

[Based on 8 live metacercariae. Metrical data for fixed material are provided in Table 6; Fig. 6c.] Body elongate-oval, 456–529 × 256–382 (490 × 328), with maximum width just anterior to ventral sucker. Oral sucker subspherical, 48–57 × 46–61 (52 × 53). Pseudosuckers elongate-oval, 69–73 × 32–40 (67 × 36). Oral opening terminal; prepharynx short; pharynx elongate-oval, 29–40 × 23–34 (35 × 26); oesophagus short, bifurcates close posterior to pharynx; caeca long, narrow, reach posterior to holdfast organ. Ventral sucker transversely oval, 54–61 × 64–71 (57 × 67), distinctly larger than oral sucker (sucker width ratio 1:1.14–1.31 (1:1.25), at mid-body length. Distance from anterior extremity of body to ventral sucker 205–265 (237). Holdfast organ large, transversely oval, bipartite, contiguous with ventral sucker, 120–158 × 152–205 (134 × 174). Excretory vesicle small, V-shaped; reserve excretory system of diplostomid type; excretory concretions predominantly medium-sized, 316–440 (372) in number, grouped into 2 lateral extracaecal [229–360 (285) excretory concretions] and 1 median [58–122 (87) excretory concretions] fields.

### Remarks

Shigin [1] suggested that the large size and number [702–854 (772)] of the excretory concretions in the metacercariae of *D. mergi* (*sensu lato*) clearly distinguish this species from all lens-infecting forms. However, molecular analyses by Georgieva et al. [7] and Selbach et al. [10] revealed the presence of at least four cryptic species within this complex. The present material is characterised by a distinctly smaller number of excretory concretions, i.e. 316–443 (372) thus adding morphological evidence to the genetic differentiation of ‘*D. mergi* Lineage 2’.

To date, ‘*D. mergi* Lineage 2’ has only been recorded/sequenced in Europe from snails in Germany: three cercarial isolates from *R. auricularia* from Hengsteysee [7] and 13 cercarial isolates from the same host in Baldeneysee, Hengsteysee and Sorpetalsperre [10]. Our study, therefore partially elucidates the life-cycle of this species, providing the first data for the second intermediate hosts in Europe comprising six new host records, all cyprinids. Similarly to the other two *Diplostomum* spp. reported here, high prevalence of infection (58%) was detected in *A. brama*. It is worth noting that a single metacercarial isolate has been sequenced from *A*. *brama* in China [6].

### *Diplostomum* sp. A

***Host*** **:** *Blicca bjoerkna* (L.) (Teleostei: Cyprinidae).

***Prevalence*** **:** 1/8 (Slovakia).

***Representative DNA sequence*** **:** KY654034 (*cox*1).

### Description

[Based on 1 fixed metacercaria; see also Table 6, Fig. 6d.] Body elongate-oval, 338 × 242, with maximum width at level of ventral sucker. Oral sucker transversely oval, 37 × 44. Pseudosuckers distinct, muscular, 47–52 long. Oral opening terminal; prepharynx absent; pharynx elongate-oval, 30 × 20; oesophagus short. Ventral sucker transversely oval, 51 × 64, larger than oral sucker (sucker width ratio 1:1.45), located at mid-body length. Distance from anterior extremity of body to ventral sucker 143. Holdfast organ small, transversely oval, bipartite, contiguous with ventral sucker, 65 × 106. Excretory vesicle small, V-shaped; reserve excretory system of diplostomid type; excretory concretions very large, 154 in number, grouped into 2 lateral extracaecal (107 excretory concretions) and 1 median (47 excretory concretions) fields. Hindbody 20 long.

### *Diplostomum* sp. B

***Host*** **:** *Carassius gibelio* (Bloch) (Teleostei: Cyprinidae).

***Prevalence*** **:** 1/6 (Slovakia).

***Representative DNA sequence*** **:** KY654035 (*cox*1).

### Description

[Based on 1 fixed metacercaria; see also Table 6, Fig. 6e.] Body elongate-oval, 426 × 304, with maximum width at level of ventral sucker. Oral sucker elongate-oval, 46 × 41. Pseudosuckers muscular, 56–58 long. Oral opening terminal; prepharynx short; pharynx elongate-oval, 41 × 22; oesophagus short, bifurcates close posterior to pharynx; caeca long, narrow, reach posterior to holdfast organ. Ventral sucker transversely oval, 51 × 59, larger than oral sucker (sucker width ratio 1:1.44), located at mid-body length. Distance from anterior extremity of body to ventral sucker 215. Holdfast organ large, transversely oval, bipartite, contiguous with ventral sucker, 115 × 136. Excretory vesicle small, V-shaped; reserve excretory system of diplostomid type; excretory concretions predominantly large, 261 in number, grouped into 2 lateral extracaecal (168 excretory concretions) and 1 median (93 excretory concretions) fields. Hindbody 19 long.

### *Diplostomum* sp. C

***Host*** **:** *Rutilus rutilus* (L.) (Teleostei: Cyprinidae).

***Prevalence*** **:** 1/8 (Slovakia).

***Representative DNA sequence*** **:** KY654036 (*cox*1).

### Description

[Based on 1 fixed metacercariae. Metrical data for the isolate are provided in Table 6; Fig. 6f.] Body oval, 381 × 278, with maximum width at level of ventral sucker. Oral sucker spherical, 47 × 47. Pseudosuckers strongly muscular, 61–67 long. Oral opening terminal; prepharynx short; pharynx 30 long. Ventral sucker transversely oval, 43 × 49, similar in size to oral sucker (sucker width ratio 1:1.04), located at mid-body length. Distance from anterior extremity of body to ventral sucker 174. Holdfast organ transversely oval, bipartite, contiguous with ventral sucker. Excretory vesicle small, V-shaped; reserve excretory system of diplostomid type; excretory concretions predominantly small, 482 in number, grouped into 2 lateral extracaecal (334 excretory concretions) and 1 median (148 excretory concretions) fields. Hindbody 16 long.

## Discussion

Parasite diversity in fishes from the River Danube has been studied extensively in the past (see Moravec [27]). However, remarkably little is known about the actual species diversity of the metacercariae of the genus *Diplostomum*. These have been typically reported as *D*. *spathaceum*, without any morphological evidence confirming species identification, or left unidentified (see Moravec [27] for details of the records). Due to the failure in achieving species identification of the metacercariae based on morphology, this practice is observed in a number of recent ecological studies of fish parasites from the River Danube (e.g. [28–32]). Recently, a single *cox*1 sequence for *D*. *pseudospathaceum* has been published from *S. glanis* in the River Danube in Romania [6].

The present study is the first taxonomically broad screening of fish hosts to provide data on the diversity of *Diplostomum* spp. from the River Danube applying molecular identification methods. The analyses based on the newly generated and published *cox*1 sequences demonstrated the presence of three species/species-level genetic lineages of *Diplostomum*, i.e. *D*. *spathaceum*, *D*. *pseudospathaceum* and ‘*D*. *mergi* Lineage 2’, and three single isolates potentially representing distinct species, i.e. *Diplostomum* spp. A-C. Our approach ensured a refined taxonomic resolution and allowed an assessment of the host ranges of the three most frequent *Diplostomum* spp. and to partly elucidate the life-cycle of one species. The large number of isolates from a wide range of hosts examined led to the detection of the somewhat higher level of mean intraspecific divergence for *D*. *spathaceum* and ‘*D*. *mergi* Lineage 2’ compared with previous data: 0.82 *vs* 0.43% [7] and 0.53% [10], and 0.47 *vs* 0% [7] and 0.30% [10], respectively.

Our novel data for host ranges of *D. spathaceum*, *D. pseudospathaceum* and ‘*D*. *mergi* Lineage 2’, based on molecular identification of the metacercariae, indicate that the transmission of these species in the River Danube is primarily associated with cyprinid fishes as second intermediate hosts. Twelve out of fourteen cyprinid species were infected with at least one species of *Diplostomum*; the largest number of species/lineages (4 out of 6) was detected in *B*. *bjoerkna. Diplostomum spathaceum* was also found in *A. ruthenus* (Acipenseridae) and *S. glanis* (Siluridae) and *D. pseudospathaceum* was recovered in *G. schraetser* (Percidae) and *Lota lota* (Lotidae). All three species of *Diplostomum* exhibited remarkably high prevalence in *A. brama*, the most well-sampled species. Although the lack of infections with *Diplostomum* spp. in 12 out of the 28 species of fish examined may be due to the small sample sizes, infections were detected in a large number of similarly under-sampled species, i.e. the acipenserid *A. ruthenus* (*D. spathaceum*), the cyprinids *A. alburnus* (‘*D*. *mergi* Lineage 2’), *B. sapa* (*D. pseudospathaceum* and ‘*D*. *mergi* Lineage 2’), *C. gibelio* (*Diplostomum* sp. B), *C. nasus* (*D. spathaceum* and ‘*D*. *mergi* Lineage 2’), *C. carpio* (*D. pseudospathaceum*), *L. aspius* (*D. spathaceum* and *D. pseudospathaceum*), *L. idus* (*D. pseudospathaceum*), *R. pigus* (*D. spathaceum*), *R. rutilus* (*D. spathaceum* and *Diplostomum* sp. C), *V. vimba* (*D. spathaceum*, *D. pseudospathaceum* and ‘*D*. *mergi* Lineage 2’), the lotid *L. lota* (*D. pseudospathaceum*), the percid *G*. *schraetser* (*D. pseudospathaceum*) and the silurid *S. glanis* (*D. spathaceum*). These data indicate that the species/lineages reported here may parasitise a wide range of hosts. The lack of specific host-related pattern of genetic structuring, illustrated by the haplotype networks for *D. spathaceum* and *D. pseudospathaceum,* based on the novel data and the pattern of shared haplotypes with isolates from fish hosts of the Cobitidae, Gasterosteidae, Percidae, Salmonidae and Siluridae (detailed in Table 3), also tend to support this suggestion. Furthermore, the apparent lack of host-specificity for *D. spathaceum* and *D. pseudospathaceum* is confirmed by the wide host ranges (17 fish species of 7 families and 12 host species of 5 families, respectively) in the expanded datasets comprising the *cox*1 sequences available to date (Figs. 3b, 4b; Additional file 2: Table S2). The most common haplotypes exhibited low host-specificity at the level of both host species (our novel data) and host family (expanded datasets).

Regarding the geographical distribution, the present comparisons with all published sequences revealed haplotypes with a wide Palaearctic distribution for two of the species, reported from Iraq and China by Locke et al. [6], i.e. *D. spathaceum* (H2: Iraq, China; H5, H7 and H10: Iraq; H13: China); ‘*D*. *mergi* Lineage 2’ (H7: China); a number of haplotypes of *D. spathaceum* (*n* = 30) are currently known from Asia only (see Locke et al. [6]; Additional file 2: Table S2).

Our study represents the first record of ‘*D*. *mergi* Lineage 2’ in a fish host in Europe and is the first to provide a morphological description of the metacercaria. The new isolates clustered together, and exhibited additional shared haplotypes, with cercarial isolates sequenced by Georgieva et al. [7] and Selbach et al. [10]. Thus, the life-cycle of this lineage was partially elucidated using molecular data, with the pulmonate snail *R*. *auricularia* acting as the first intermediate host and six cyprinid fishes (*A*. *alburnus*, *A*. *brama*, *B*. *bjoerkna*, *B*. *sapa*, *C*. *nasus* and *V*. *vimba*) acting as second intermediate hosts. The cercaria of ‘*D*. *mergi* Lineage 2’ was described in detail by Selbach et al. [10] who differentiated it from the cercaria of *D. mergi* *sensu* Niewiadomska & Kiselienė, 1994 [33] by having furcae longer than the tail stem and by morphometry, and from the cercariae of the four species within the “*D*. *mergi*” species complex by five unique morphometric features (see Selbach et al. [10] for details). The present metacercariae exhibited markedly smaller number of excretory concretions in comparison with the metacercariae of *D. mergi* (*sensu* *lato*) (mean 372 *vs* 772; see [1]) and showed morphometric differences from the metacercariae of the other lens-infecting species, *D. spathaceum* and *D. pseudospathaceum.* These data, in association with the genetic evidence, support the distinct species status of ‘*D. mergi* Lineage 2’; however, formal description of the species would require the discovery of the adult stage. The distribution of this species-level genetic lineage is apparently wider, and not restricted to Europe, since Locke et al. [6] reported a single sequence from a metacercaria in the cyprinid *A*. *brama* from China. Further studies would add to our knowledge of haplotype diversity, host ranges and geographical distribution of this lineage.

Brabec et al. [25] characterised the complete mitochondrial genomes of the two closely related species, *D*. *spathaceum* and *D*. *pseudospathaceum* and carried out a comparative genome assessment. These authors revealed that the *cox*1 gene and its ‘barcode’ region, currently applied for molecular identification, represent the most conserved protein-coding regions of the mitochondrial genome of *Diplostomum* spp. and identified *nad*4 and *nad*5 genes as most promising molecular diagnostic markers. In the pilot *nad* gene sequencing carried out here, we opted for *nad*3 gene, a slightly more conserved in comparison to the *nad*4 and *nad*5 genes, because the identification based on *cox*1 revealed the presence of a lineage of the “*D. mergi*” species complex that was shown to be rather distant to the two sibling species studied by Brabec et al. [25] (e.g. [7, 10]). Our results indicate that the newly designed primers can be used for successful amplification of *nad*3 within the “*D. mergi*” complex and possibly in other distantly related lineages of *Diplostomum*; the markedly higher levels of interspecific divergence compared to *cox*1 indicate that the *nad*3 gene is a good candidate marker for multi-gene approaches to systematic estimates within the genus.

## Conclusions

The first exploration of the species diversity and host ranges of *Diplostomum* spp., based on molecular and morphological evidence from a broad range of fish hosts in the River Danube (Hungary and Slovakia), revealed the presence of three species/species-level genetic lineages of *Diplostomum*, i.e. *D*. *spathaceum*, *D*. *pseudospathaceum* and ‘*D*. *mergi* Lineage 2’, and three single isolates potentially representing distinct species. The most frequently found *Diplostomum* spp. exhibited a low host-specificity, predominantly infecting a wide range of cyprinid fishes but also species of distant fish families such as the Acipenseridae, Lotidae, Percidae and Siluridae. Our study provided the first *cox*1 and *nad*3 sequences associated with a morphological characterisation for metacercariae of ‘*D*. *mergi* Lineage 2’ in a fish host in Europe and partially elucidated the life-cycle of this species using molecular data. The novel sequence data will advance further ecological studies on the distribution and host ranges of these important fish parasites in Europe.

## Additional files

Additional file 1: Table S1. Summary data for the sequences from isolates of *Diplostomum* spp. isolates retrieved from the GenBank database and used in the phylogenetic analyses. (DOC 67 kb)

Additional file 2: Table S2. Summary data for the sequences for *Diplostomum spathaceum* and *D*. *pseudospathaceum* from metacercarial isolates used in the expanded haplotype networks. (DOCX 31 kb)
