# Supplementary material for: Exploring the diversity of Diplostomum (Digenea: Diplostomidae) in fishes from the River Danube using mitochondrial DNA barcodes
Source: Parasit Vectors. 2017 Dec 2;10:592. doi: 10.1186/s13071-017-2518-5 (PMC5712130; doi:10.1186/s13071-017-2518-5)
Supplement: Supplementary file 1 — Summary data for the sequences from isolates of Diplostomum spp. isolates retrieved from the GenBank database and used in the phylogenetic analyses. (DOC 67 kb) [file 13071_2017_2518_MOESM1_ESM.doc]

**Additional file 1: Table S1.** Summary data for the sequences from isolates of *Diplostomum* spp. retrieved from the GenBank database and used in the phylogenetic analyses

| **Species** | **Host** | **Isolate** | **GenBank accession number** | **Source** |
| --- | --- | --- | --- | --- |
| ‘*Diplostomum baeri*’ 1^a^ | *Salmo trutta fario* | STR4 | JX986864 | Georgieva et al. [1] |
| ‘*Diplostomum baeri*’ 1^a^ | *Salmo trutta fario* | STL2 | JX986865 | Georgieva et al. [1] |
| ‘*Diplostomum baeri*’ 1^a^ | *Salmo trutta fario* | STR7 | JX986869 | Georgieva et al. [1] |
| ‘*Diplostomum baeri*’ 2^b^ | *Perca fluviatilis* | PF8D7 | JQ639191 | Behrmann-Godel [2] |
| ‘*Diplostomum baeri*’ 2^b^ | *Perca fluviatilis* | PF15D9 | JQ639193 | Behrmann-Godel [2] |
| ‘*Diplostomum baeri*’ 2^b^ | *Perca fluviatilis* | PF5D3 | JQ639195 | Behrmann-Godel [2] |
| *Diplostomum huronense* | *Catostomus commersoni* | D.RL.D.Cc.1.5 | GQ292490 | Locke et al. [3] |
| *Diplostomum huronense* | Exp. infection | D.IN.1.G.L35W.6 | FJ477197 | Moszczynska et al. [4] |
| *Diplostomum parviventosum*^a^ | *Radix auricularia* | RaHe1 | KR149504 | Selbach et al. [5] |
| *Diplostomum parviventosum*^a^ | *Radix auricularia* | RaHe2 | KR149505 | Selbach et al. [5] |
| *Diplostomum parviventosum*^a^ | *Radix auricularia* | RaHe3 | KR149506 | Selbach et al. [5] |
| ‘*Diplostomum mergi* 2’^a^ | *Radix auricularia* | RAH2 | JX986874 | Georgieva et al. [1] |
| ‘*Diplostomum mergi* 2’^a^ | *Radix auricularia* | RaHe11 | KR149515 | Selbach et al. [5] |
| ‘*Diplostomum mergi* 2’^a^ | *Radix auricularia* | RaBa1 | KR149513 | Selbach et al. [5] |
| ‘*Diplostomum mergi* 3’^a^ | *Radix auricularia* | RaHe16 | KR149524 | Selbach et al. [5] |
| ‘*Diplostomum mergi* 3’^a^ | *Radix auricularia* | RaHe18 | KR149526 | Selbach et al. [5] |
| ‘*Diplostomum mergi* 3’^a^ | *Salmo trutta fario* | STR13 | JX986881 | Georgieva et al. [1] |
| ‘*Diplostomum mergi* 3’^a^ | *Gobio gobio* | GGR4 | JX986884 | Georgieva et al. [1] |
| ‘*Diplostomum mergi* 3’^a^ | *Salmo trutta fario* | STR15 | JX986886 | Georgieva et al. [1] |
| ‘*Diplostomum mergi* 4’^a^ | *Radix auricularia* | RaHe20 | KR149528 | Selbach et al. [5] |
| *Diplostomum spathaceum* | *Larus argentatus michahellis* | LCED3 | KP025774 | Pérez-del-Olmo et al. [6] |
| *Diplostomum spathaceum* | *Pseudochondrostoma willkommii* | PWVG5 | KP025784 | Pérez-del-Olmo et al. [6] |
| *Diplostomum spathaceum* | *Gasterosteus aculeatus* | GAH3 | JX986893 | Georgieva et al. [1] |
| *Diplostomum spathaceum* | *Larus ridibundus* | spa3a | KR269763^c^ | Brabec et al. [7] |
| *Diplostomum pseudospathaceum* | *Lymnaea stagnalis* | LSB3 | JX986907 | Georgieva et al. [1] |
| *Diplostomum pseudospathaceum* | *Larus argentatus* | LAG2 | JX986904 | Georgieva et al. [1] |
| *Diplostomum pseudospathaceum* | *Lymnaea stagnalis* | LSB2 | JX986899 | Georgieva et al. [1] |
| *Diplostomum pseudospathaceum* | *Larus ridibundus* | pse3a | KR269764^c^ | Brabec et al. [7] |
| ‘*Diplostomum* sp. Clade Q’^b^ | *Cyprinus carpio* | CCED | KP025770 | Pérez-del-Olmo et al. [6] |
| ‘*Diplostomum* sp. Clade Q’^b^ | *Radix auricularia* | RaHe28 | KR149554 | Selbach et al. [5] |
| ‘*Diplostomum* sp. Clade Q’^b^ | *Rutilus rutilus* | RR43 | JQ639177 | Behrmann-Godel [2] |
| ‘*Diplostomum* sp. Clade Q’^b^ | *Rutilus rutilus* | RR45 | JQ639178 | Behrmann-Godel [2] |
| ‘*Diplostomum* sp. Clade Q’^b^ | *Radix auricularia* | RA97 | JQ639179 | Behrmann-Godel [2] |
| *Tylodelphys clavata* (outgroup) | *Perca fluviatilis* | PFL1 | JX986909 | Georgieva et al. [1] |

^a^Species/lineages discovered in the River Rurh drainage, Germany, and characterised molecularly and morphologically by Georgieva et al. [7] and Selbach et al. [10]

^b^*sensu* Georgieva et al. [7]

^c^*nad*3 only

**References**

1. Georgieva S, Soldánová M, Pérez-del-Olmo A, Dangel RD, Sitko J, Sures B, et al. Molecular prospecting for European *Diplostomum* (Digenea: Diplostomidae) reveals cryptic diversity. Int J Parasitol. 2013;43:57–72.

2. Behrmann-Godel J. Parasite identification, succession and infection pathways in perch fry (*Perca fluviatilis*): new insights through a combined morphological and genetic approach. Parasitology. 2013;140:509–520.

3. Locke SA, McLaughlin JD, Dayanandan S, Marcogliese DJ. Diversity, specificity and evidence of hybridization in *Diplostomum* spp. metacercariae in freshwater fishes is revealed by DNA barcodes and ITS sequences. Int J Parasitol. 2010a;40:333–43.

4. Moszczynska A, Locke SA, McLaughlin JD, Marcogliese DJ, Crease TJ. Development of primers for the mitochondrial cytochrome *c* oxidase I gene in digenetic trematodes illustrates the challenge of barcoding parasitic helminths. Mol Ecol Resour. 2009;9:75–82.

5. Selbach C, Soldánová M, Georgieva S, Kostadinova A, Sures B. Integrative taxonomic approach to the cryptic diversity of *Diplostomum* spp. in lymnaeid snails from Europe with a focus on the ‘*Diplostomum mergi*’ species complex. Parasit Vectors. 2015;8:300.

6. Pérez-del-Olmo A, Georgieva S, Pula HJ, Kostadinova A. Molecular and morphological evidence for three species of *Diplostomum* (Digenea: Diplostomidae), parasites of fishes and fish-eating birds in Spain. Parasit Vectors. 2014;7:502.

7. Brabec J, Kostadinova A, Scholz T, Littlewood DT. Complete mitochondrial genomes and nuclear ribosomal RNA operons of two species of *Diplostomum* (Platyhelminthes: Trematoda): a molecular resource for taxonomy and molecular epidemiology of important fish pathogens. Parasit Vectors. 2015;8:336.
