# Supplementary material for: Exploring the diversity of Diplostomum (Digenea: Diplostomidae) in fishes from the River Danube using mitochondrial DNA barcodes
Source: Parasit Vectors. 2017 Dec 2;10:592. doi: 10.1186/s13071-017-2518-5 (PMC5712130; doi:10.1186/s13071-017-2518-5)
Supplement: Supplementary file 2 — Summary data for the sequences for Diplostomum spathaceum and D. pseudospathaceum from metacercarial isolates used in the expanded haplotype networks. (DOCX 31 kb) [file 13071_2017_2518_MOESM2_ESM.docx]

**Additional file 2: Table S2.** Summary data for the sequences for *Diplostomum* *spathaceum* and *D*. *pseudospathaceum* from metacercarial isolates used in the expanded haplotype networks

| **Species/ Haplotype** | **GenBank ID** | **Host** | **Origin** | **Reference** |
| --- | --- | --- | --- | --- |
| ***Diplostomum spathaceum*** | |  |  |  |
| H1 | KY653962, KY653963; KY653982; KY653985, KY653986 | *Abramis brama*; *Rutilus rutilus*; *Vimba vimba* | Slovakia | Present study |
| H2 | KY653968, KR271430; KR271451; KR271463; KR271426; JX986888, KJ726433, KJ726434; KY653977; KY653979 | *Abramis brama*; *Acanthobrama marmid*; *Carasobarbus luteus*^a^; *Cyprinion macrostomum*; *Gasterosteus aculeatus*; *Leuciscus aspius*; *Rutilus pigus* | China; Germany; Iceland; Iraq; Slovakia | Locke et al. [1]; Georgieva et al. [2]; Blasco-Costa et al. [3]; Present study |
| H3 | KY653969; JX986894; KR271417; KY653981; KY653984 | *Abramis brama*; *Gasterosteus aculeatus*; *Perca fluviatilis*; *Rutilus pigus*; *Silurus glanis* | Germany; Italy; Slovakia | Georgieva et al. [2]; Locke et al. [1]; Present study |
| H4 | KY653970; KY653972; JX986893; KP025785; KJ726438; KR271462 | *Acipenser ruthenus*; *Blicca bjoerkna*; *Gasterosteus aculeatus*; *Pseudochondrostoma willkommii*; *Salvelinus alpinus*; *Silurus glanis* | Germany; Iceland; Romania; Slovakia; Spain | Georgieva et al. [2]; Pérez-del-Olmo et al. [4]; Blasco-Costa et al. [3]; Locke et al. [1]; Present study |
| H5 | KY653964; KR271422, KR271429; KP025783; KY653978 | *Abramis brama*; *Cyprinion macrostomum*; *Pseudochondrostoma willkommii*; *Rutilus pigus* | Iraq; Slovakia; Spain | Locke et al. [1]; Pérez-del-Olmo et al. [4]; Present study |
| H6 | KY653971; KJ726435; KJ726436; KP025778; KP025781 | *Blicca bjoerkna*; *Gasterosteus aculeatus*; *Misgurnus anguillicaudatus*; *Pseudochondrostoma willkommii* | Iceland; Slovakia; Spain | Blasco-Costa et al. [3]; Pérez-del-Olmo et al. [4]; Present study |
| H7 | KR271423; KY653974; KR271452; JX986890; KP025782, KP025786 | *Acanthobrama marmid*; *Chondrostoma nasus*; *Cyprinion macrostomum*; *Gasterosteus aculeatus*; *Pseudochondrostoma willkommii* | Germany; Iraq; Slovakia, Spain | Georgieva et al. [2]; Locke et al. [1]; Pérez-del-Olmo et al. [4]; Present study |
| H8 | KY653980 | *Rutilus pigus* | Slovakia | Present study |
| H9 | KY653965 | *Abramis brama* | Slovakia | Present study |
| H10 | KY653967; KR271428; KP025779 | *Abramis brama*; *Carasobarbus luteus*^a^; *Misgurnus anguillicaudatus* | Iraq; Slovakia; Spain | Locke et al. [1]; Pérez-del-Olmo et al. [4]; Present study |
| H11 | KY653961 | *Abramis brama* | Slovakia | Present study |
| H12 | KY653966; KR271420 | *Abramis brama*; *Perca fluviatilis* | Italy; Slovakia | Locke et al. [1]; Present study |
| H13 | KR271459; KY653976 | *Abramis brama*; *Leuciscus aspius* | China; Slovakia | Locke et al. [1]; Present study |
| H14 | KY653973 | *Blicca bjoerkna* | Slovakia | Present study |
| H15 | KY653975 | *Chondrostoma nasus* | Slovakia | Present study |
| H16 | KY653983 | *Rutilus rutilus* | Slovakia | Present study |
| H17 | KP025780 | *Pseudochondrostoma willkommii* | Spain | Pérez-del-Olmo et al. [4] |
| H18 | KP025784 | *Pseudochondrostoma willkommii* | Spain | Pérez-del-Olmo et al. [4] |
| H19 | KP025787 | *Silurus glanis* | Spain | Pérez-del-Olmo et al. [4] |
| H20 | KJ726437 | *Gasterosteus aculeatus* | Iceland | Blasco-Costa et al. [3] |
| H21 | KJ726439 | *Gasterosteus aculeatus* | Iceland | Blasco-Costa et al. [3] |
| H22 | KR271468 | *Perca fluviatilis* | Italy | Locke et al. [1] |
| H23 | KR271457 | *Perca fluviatilis* | Italy | Locke et al. [1] |
| H24 | KR271442 | *Silurus glanis* | Romania | Locke et al. [1] |
| H25 | KR271445 | *Rutilus rutilus* | Romania | Locke et al. [1] |
| H26 | KR271433 | *Abramis brama* | China | Locke et al. [1] |
| H27 | KR271464 | *Carasobarbus luteus*^a^ | Iraq | Locke et al. [1] |
| H28 | KR271419 | *Cyprinion macrostomum* | Iraq | Locke et al. [1] |
| H29 | KR271446 | *Abramis brama* | China | Locke et al. [1] |
| H30 | KR271441 | *Acanthobrama marmid* | Iraq | Locke et al. [1] |
| H31 | KR271432 | *Carasobarbus luteus*^a^ | Iraq | Locke et al. [1] |
| H32 | KR271456 | *Acanthobrama marmid* | Iraq | Locke et al. [1] |
| H33 | KR271460 | *Cyprinion macrostomum* | Iraq | Locke et al. [1] |
| H34 | KR271467 | *Acanthobrama marmid* | Iraq | Locke et al. [1] |
| H35 | KR271469 | *Carasobarbus luteus*^a^ | Iraq | Locke et al. [1] |
| H36 | KR271455 | *Acanthobrama marmid* | Iraq | Locke et al. [1] |
| H37 | KR271436 | *Carasobarbus luteus*^a^ | Iraq | Locke et al. [1] |
| H38 | KR271434 | *Abramis brama* | China | Locke et al. [1] |
| H39 | KR271454 | *Acanthobrama marmid* | Iraq | Locke et al. [1] |
| H40 | KR271424 | *Alburnus caeruleus* | Iraq | Locke et al. [1] |
| H41 | KR271427 | *Cyprinion macrostomum* | Iraq | Locke et al. [1] |
| H42 | KR271438 | *Cyprinion macrostomum* | Iraq | Locke et al. [1] |
| H43 | KR271465 | *Carasobarbus luteus*^a^ | Iraq | Locke et al. [1] |
| H44 | KR271449 | *Cyprinion macrostomum* | Iraq | Locke et al. [1] |
| H45 | KR271453 | *Cyprinion macrostomum* | Iraq | Locke et al. [1] |
| H46 | KR271435 | *Acanthobrama marmid* | Iraq | Locke et al. [1] |
| H47 | KR271447 | *Cyprinion macrostomum* | Iraq | Locke et al. [1] |
| H48 | KR271444 | *Acanthobrama marmid* | Iraq | Locke et al. [1] |
| H49 | KR271461 | *Cyprinion macrostomum* | Iraq | Locke et al. [1] |
| H50 | KR271440 | *Cyprinion macrostomum* | Iraq | Locke et al. [1] |
| H51 | KR271416 | *Acanthobrama marmid* | Iraq | Locke et al. [1] |
| H52 | KR271415 | *Acanthobrama marmid* | Iraq | Locke et al. [1] |
| H53 | KR271443 | *Abramis brama* | China | Locke et al. [1] |
| H54 | KR271439 | *Cyprinion macrostomum* | Iraq | Locke et al. [1] |
| H55 | KR271421 | *Acanthobrama marmid* | Iraq | Locke et al. [1] |
|  |  |  |  |  |
| ***Diplostomum pseudospathaceum*** | |  |  |  |
| H1 | KY653987, KY653988; KY653992; KY653996; KY654003; KY654006; KY654012; KR271090, KR271091; KY654015, KY654016 | *Abramis brama*; *Ballerus* *sapa*; *Blicca bjoerkna*; *Cyprinus carpio*; *Leuciscus aspius*; *Leuciscus idus*; *Silurus glanis*; *Vimba vimba* | Romania; Slovakia | Locke et al. [1]; Present study |
| H2 | KY653989; KY653995; KR271093; KY654007 | *Abramis brama*; *Ballerus sapa*; *Cyprinus carpio*; *Leuciscus aspius* | Romania; Slovakia | Locke et al. [1]; Present study |
| H3 | KY653993, KY653994; KY654014 | *Ballerus sapa*; *Lota lota* | Slovakia | Present study |
| H4 | KY654001; KY654004; KY654011 | *Blicca bjoerkna*; *Gymnocephalus schraetsor*; *Leuciscus aspius* | Slovakia | Present study |
| H5 | JX986902, JX986903; KY654009, KY654010 | *Gasterosteus aculeatus*; *Leuciscus aspius* | Germany; Slovakia | Georgieva et al. [2]; Present study |
| H6 | KY654008 | *Leuciscus aspius* | Slovakia | Present study |
| H7 | KY653997 | *Blicca bjoerkna* | Slovakia | Present study |
| H8 | KY653998 | *Blicca bjoerkna* | Slovakia | Present study |
| H9 | KY654002 | *Blicca bjoerkna* | Slovakia | Present study |
| H10 | KY653999 | *Blicca bjoerkna* | Slovakia | Present study |
| H11 | KY654000 | *Blicca bjoerkna* | Slovakia | Present study |
| H12 | KY654013 | *Leuciscus idus* | Slovakia | Present study |
| H13 | KY654005 | *Leuciscus aspius* | Slovakia | Present study |
| H14 | KY653990 | *Abramis brama* | Slovakia | Present study |
| H15 | KY653991 | *Abramis brama* | Slovakia | Present study |
| H16 | KR271085 | *Silurus glanis* | Romania | Locke et al. [1] |
| H17 | KR271089 | *Silurus glanis* | Romania | Locke et al. [1] |
| H18 | KR271084 | *Silurus glanis* | Romania | Locke et al. [1] |
| H19 | KR271092 | *Silurus glanis* | Romania | Locke et al. [1] |
| H20 | KR271086 | *Silurus glanis* | Romania | Locke et al. [1] |
| H21 | KR271083 | *Rutilus rutilus* | Romania | Locke et al. [1] |

Six sequences for *D*. *spathaceum* (KR271418, KR271425, KR271448, KR271458, KR271450 and KR271466) and a single sequence for *D*. *pseudospathaceum* (KR271094) published by Locke et al. [1] were too short and thus not considered in haplotype identification

^a^Reported as *Barbus luteus* [1]

**References**

1. Locke SA, Al-Nasiri FS, Caffara M, Drago F, Kalbe M, Lapierre AR, et al. Diversity, specificity and speciation in larval Diplostomidae (Platyhelminthes: Digenea) in the eyes of freshwater fish, as revealed by DNA barcodes. Int J Parasitol. 2015;45:841–855.

2. Georgieva S, Soldánová M, Pérez-del-Olmo A, Dangel RD, Sitko J, Sures B, et al. Molecular prospecting for European *Diplostomum* (Digenea: Diplostomidae) reveals cryptic diversity. Int J Parasitol. 2013;43:57–72.

3. Blasco-Costa I, Faltýnková A, Georgieva S, Skírnisson K, Scholz T, Kostadinova A. Fish pathogens near the Arctic Circle: molecular, morphological and ecological evidence for unexpected diversity of *Diplostomum* (Digenea: Diplostomidae) in Iceland. Int J Parasitol. 2014;44:703–715.

4. Pérez-del-Olmo A, Georgieva S, Pula HJ, Kostadinova A. Molecular and morphological evidence for three species of *Diplostomum* (Digenea: Diplostomidae), parasites of fishes and fish-eating birds in Spain. Parasit Vectors. 2014;7:502.
